# Isolation of Two Strong Poly (U) Binding Proteins from Moderate Halophile *Halomonas eurihalina* and Their Identification as Cold Shock Proteins

**DOI:** 10.1371/journal.pone.0034409

**Published:** 2012-04-13

**Authors:** Usha kumari Garapati, Tangirala Suryanarayana

**Affiliations:** Department of Biochemistry, School of Life Sciences, University of Hyderabad, Hyderabad, India; Louisiana State University and A & M College, United States of America

## Abstract

Cold shock proteins (Csp) are known to be expressed in response to sudden decrease in temperature. They are thought to be involved in a number of cellular processes viz., RNA chaperone activity, translation, transcription, nucleoid condensation. During our studies on ribosomal protein S1 in moderate halophile *Halomonas eurihalina*, we observed the presence of two strong poly (U) binding proteins in abundance in cell extracts from cells grown under normal growth conditions. The proteins can be isolated in a single step using Poly (U) cellulose chromatography. The proteins were identified as major cold shock proteins belonging to Csp A family by MALDI-TOF and bioinformatic analysis. Csp 12 kDa was found in both exponential and stationary phases whereas Csp 8 kDa is found only in exponential phase.

## Introduction

Cold shock proteins (CSPs) were known to be induced by a sudden downward shift in temperature [1]–[4] and these cold shock proteins are conserved from archaea to eukaryotes. In the literature, it was reported that *Escherichia coli* cold shock proteins are of two types. Class I proteins are expressed at extremely low levels at 37°C and their levels increased dramatically after exposure to low temperature. CspA, CspB, CspG, CsdA etc. are reported to be class I proteins. Class II proteins are present under normal growth conditions but their levels increased by about 10 fold after cold shock treatment. Examples of class II proteins include IF-2, RecA, H-NS etc. [3], [4]. Among these, CspA, CspB and CspG were known as major cold shock proteins due to their abundance under cold shock conditions [5]. However, it was reported that the designation of CspA as a major cold shock protein as misnomer because of its abundance in early exponential growth at 37(C in E. coli cells that were never exposed to cold shock. It was also reported that the levels of CspA were almost undetectable in mid-to-late exponential growth phases [6]. In spite of extensive research carried out on CspA and other cold shock proteins, their physiological role remains elusive. However, CspA has been implicated in RNA/DNA chaperone activity by various research groups [7]–[9]. Structural and functional properties of cold shock proteins from archaea have also been reported [10]. Structural, molecular and physiological studies on different cold shock proteins have been reviewed [11]–[13].

In our laboratory work is being carried out on ribosomes from H. eurihalina. During our studies, we performed poly (U) cellulose chromatography of post-ribosomal supernatant fraction (S-100) to detect ribosomal protein S1, which has strong affinity to polypyrimidines [14], [15]. However, Poly (U) cellulose chromatography revealed presence of two low molecular weight proteins (12(kDa and 8(kDa) with high affinity to poly (U) which were identified as major cold shock proteins. We report here that even in the absence of cold shock, major cold shock proteins are expressed in abundance in both exponential and stationary phases of growth in this moderate halophilic organism. Our results suggest that major cold shock proteins may have a function in normal cellular metabolism and in protection of cells from other stress conditions such as salt stress as in the case of H. eurihalina.

## Materials and Methods

### Bacterial strain and growth conditions

The moderate halophile Halomonas eurihalina DSM 5720 was obtained from DSMZ GmbH (German Collection of Microorganisms and cell cultures), Braunschweig, Germany. H. eurihalina cells were grown in an orbital shaker at 30°C in moderate halophile (MH) medium (pH was adjusted to 7.2 with 2N NaOH) containing 6% NaCl, 1.5% MgCl_2_, 0.74% MgSO_4_, 0.027% CaCl_2_, 0.15% KCl, 0.0045% NaHCO_3_, 0.0019% NaBr, 0.5% peptone, 1% yeast extract, 0.1% glucose with shaking at 100 cycles per minute as recommended by DSMZ, Germany. The cultures after 12 hrs (OD_660_ 0.8) and 24 hrs (OD_660_ 2.0) of growth for exponential and stationary phase cells respectively were chilled and harvested at 4°C.

### Preparation of S-100

Post-ribosomal supernatant fraction (S-100) and ribosomes from exponential and stationary phase *H. eurihalina* cells (20 grams) were prepared by grinding with alumina as previously described by Minks *et al.* 1978 [16]. Alumina ground cell extracts were prepared in buffer containing 20 mM Tris-pH 7.6, 250 mM ammonium chloride, 20 mM magnesium acetate and 7 mM 2-mercaptoethanol (TNM_20_Me)

### Coupling of poly (U) to cellulose and poly (U) cellulose chromatography

Poly (U) was coupled to activated cellulose according to Carmichael, 1975 [14]. Small columns were packed with poly (U)-cellulose (approx. 5 ml bed volume). Exponential and stationary phase S-100 samples (6 ml each) were loaded separately on these columns previously equilibrated with TNM_20_Me buffer. Each column was washed with 20 ml of buffer containing 20 mM Tris-Cl pH 7.6, 20 mM magnesium acetate, 1 M ammonium chloride and 7 mM 2-mercaptoethanol (buffer A) at a flow rate of 20 ml/h to remove non-specific proteins. Immediately, the columns were eluted with 15 ml of buffer A containing 8 M urea. Chromatography was performed at 4°C.

### SDS-PAGE and Two-dimensional gel electrophoresis

Fractions showing absorbance at 280 nm were analyzed by SDS-PAGE. For better resolution of low molecular weight proteins 18% SDS-PAGE was performed according to Thomas and Kornberg [17]. Prestained protein molecular mass-standards (10 to 170 kDa, Fermentas) were used. For two dimensional polyacrylamide gel electrophoresis, the fractions were precipitated with 5 volumes of ice cold acetone overnight at −20°C and centrifuged at 10000 g for 30 min to remove salts. Protein concentration was determined by using Bradford reagent (Sigma-Aldrich chemicals, USA) using bovine serum albumin as standard. Protein samples (30 µg for each gel) were suspended in 6 M urea, 10 mM DTT and 20 mM Bis-Tris acetic acid and 5 mM MES buffer. First dimensional gel electrophoresis was carried out according to system I of Madjar *et al.* [18] without any modifications. Electrophoresis in the second dimension was carried out exactly as described by Metz and Bogorad [19]. One dimensional slab gel electrophoresis was carried out under non denaturing conditions according to SDS-PAGE system of Laemmli [20] without SDS in gel and electrode buffer.

### Proteomic analysis: in-gel digestion and mass spectrometry (MS)

In-gel digestion and matrix-assisted laser desorption/ionization time of flight mass spectrometric (MALDI-TOF MS) analysis was conducted with a MALDI-TOF/TOF mass spectrometer (Bruker Autoflex III smartbeam, Bruker Daltonics, Bremen, Germany) according to the method described by Shevchenko *et al.*1996 [21] with slight modifications. Coomassie-stained protein spots were manually excised from three reproducible gels. The excised gel pieces were destained with 100 µL of 50% acetonitrile (ACN) in 25 mM ammonium bicarbonate (NH_4_HCO_3_) for five times. Thereafter, the gel pieces were treated with 10 mM DTT in 25 mM NH_4_HCO_3_ and incubated at 56°C for 1 h. This is followed by treatment with 55 mM iodoacetamide in 25 mM NH_4_HCO_3_ for 45 min at room temperature (25±2°C), washed with 25 mM NH_4_HCO_3_ and ACN, dried in speed vac concentrator and rehydrated in 20 µL of 25 mM NH_4_HCO_3_ solution containing 12.5 ng µL^−1^ trypsin (sequencing grade, Promega, Wisconsin, USA). The above mixture was incubated on ice for 10 min and kept overnight for digestion at 37°C. After digestion, a short spin for 10 min was given and the supernatant was collected in a fresh Eppendorf tube. The gel pieces were re-extracted with 50 µL of 1% trifluoroacetic acid (TFA) and ACN (1∶1) for 15 min with frequent vortexing. The supernatants were pooled and dried using speed vac concentrator and the residue was reconstituted in 5 µL of 1∶1 ACN and 1% TFA. 2 µL of the above sample was mixed with 2 µL of freshly prepared α-cyano-4-hydroxycinnamic acid (CHCA) matrix in 50% ACN and 1% TFA (1∶1) and 1 µL was spotted on target plate.

### Protein identification: peptide mass fingerprinting and MS/MS analysis

Protein identification was performed by database searches (PMF and MS/MS) using MASCOT program (http://www.matrixscience.com/) employing Biotools software (Bruker Daltonics).The similarity search for mass values was done with existing digests and sequence information from NCBInr and Swiss-Prot database. The taxonomic categories searched were eubacteria, archaea (archaebacteria) and eukaryotes. The other search parameters were: fixed modification of carbamidomethyl (C), variable modification of oxidation (M), enzyme trypsin, peptide charge of 1^+^ and monoisotopic. According to the MASCOT probability analysis (P\0.05), only significant hits were accepted for protein identification. BLAST (http://blast.ncbi.nlm.nih.gov/Blast.cgi) was used for sequence homology searches and detection of conserved domains in the peptide sequences obtained by MALDI-TOF MS analysis. These data were submitted as supplementary material (Supporting information: File S1, and File S2).

## Results and Discussion

**Figure 1:**
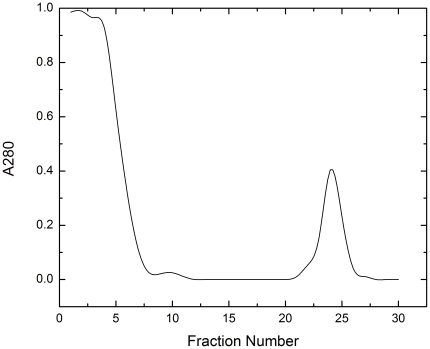
Elution profile of S-100 on poly (U)-cellulose column. Fractions 1–20 were eluted with buffer A. Fractions 21–30 eluted with buffer B. Absorbance at 280 nm for the fractions eluted with buffer A shows the release of non-specific proteins bound to the poly (U) column. Single, sharp peak represents the fractions containing eluted poly (U) binding proteins.

Present work was carried out to detect the presence of ribosomal protein S1 in post ribosomal supernatant (S-100) of *H. eurihalina*, which has very strong affinity to poly (U). Chromatographic elution profile of S-100 from exponential and stationary phase cells on poly (U)-cellulose column with buffer B showed single, sharp peak (Fig. 1). The requirement of buffer containing 8 M urea and 1 M NH_4_Cl indicated strong affinity of these proteins to poly (U), which fail to dissociate in presence of buffers containing 1 M NH_4_Cl and urea less than 8 M. Bound fraction was analyzed by different electrophoretic methods.

**Figure 2:**
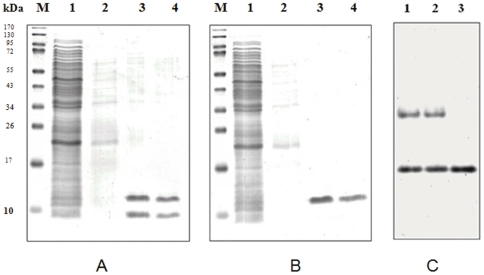
Poly acrylamide gel electrophoretic analysis of poly (U) cellulose chromatography peak fractions. A. SDS-PAGE of fractions from exponential phase S-100. B. SDS-PAGE of fractions from stationary phase S-100. In both cases, Lane M: Molecular weight marker, lane 1: exponential/stationary phase S-100 (10 µl), lane 2: fraction number 3 (100 µl) and lanes 3–4: fractions 24 and 25 (100 µl each). C. Native-PAGE analysis. Lane 1–2: Fractions 24 and 25 of exponential phase S-100 elution, lane 3: Fraction 24 of stationary phase S-100 elution.

**Figure 3:**
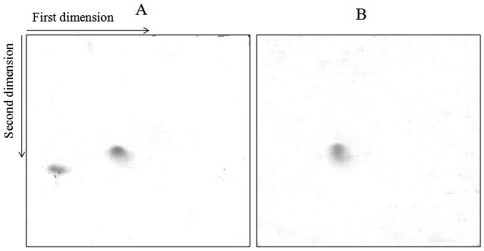
Two-dimensional gel electrophoresis of poly (U) binding proteins isolated from S-100. A. Exponential phase. B. Stationary phase. Electrophoresis was performed as described under Methods.

**Electrophoretic analysis of proteins strongly bound to Poly (U) cellulose: SDS-PAGE** analysis showed that even though elution profile was similar, exponential phase fractions contained two low molecular weight proteins (12 kDa and 8 kDa) where as stationary phase fractions contained one protein (12 kDa) (Fig. 2A & B) and no ribosomal protein S1 was detected. Electrophoresis of the fractions in the absence of SDS showed that the proteins are acidic and that the 12 kDa protein, which is present in both exponential and stationary phase, is more acidic than the 8 kDa protein (Fig. 2C). Two-dimensional gel electrophoresis of the proteins resulted in separation of both the proteins depending on their acidity and molecular weight (Fig. 3A & 3B). Thus poly (U)-cellulose chromatography can be used to purify cold shock proteins in a single step. It should also be noted that elution of column at a low flow rate with at least 4 to 5 bed volumes of buffer A significantly decreases the presence of non-specific proteins.

### Identification of the proteins by MALDI-TOF MS analysis and BLAST search

For identification, the 12 kDa protein was named as spot number 1 and 8 kDa protein as spot number 2. Spot number, precursor ion mass, matched peptide sequence, source organism, accession number, protein ID, experimental and theoretical molecular weight and MS/MS ion score of both the proteins were shown in Table 1. The peptide sequences thus obtained were used to detect the presence of any conserved domains and sequence homologies. Conserved domain CSP_CSD (accession number: cd04458), a S1-like cold shock domain (OB fold) was detected for the peptide sequence of 12 kDa protein by using BLAST search. Conserved domain CSD (accession number: pfam00313) ‘cold shock’ DNA binding domain was detected for the peptide sequence of 8 kDa protein. CSD (cold shock domain) consists of ∼70 amino acids and is highly conserved from prokaryotes to humans. CSD harbors RNP1 and RNP2, the nucleic acid binding motifs. Cold shock proteins are the subgroup of CSD superfamily proteins found in bacteria [22]. The sequence homology for the detected conserved domains is significant as the entire peptides were found to be homologous to the domain. Both the peptide sequences were found to be more homologous to CspA family than other CSPs during BLAST search. Additionally, bioinformatic studies were performed using matched peptides of 12 kDa and 8 kDa for blast search. The results obtained showed several hundreds of hits for cold shock protein or CSD proteins from different organisms. Majority of the top hundred hits for 12 kDa protein showed homology to CspA family proteins and for 8 kDa protein showed homology to cold shock-DNA binding domain-containing protein respectively. We have not performed multiple sequence alignments for all these hits because the results would be voluminous. However, both the peptides were used to blast search KEGG genome data base of *Halomonas elongata* which gave four hits for CspA cold shock proteins from *H. elongata*. Three of these proteins (all 68 amino acid residues) were HELO_3240 (NCBI-gene ID 9746494), HELO_3431 (NCB-gene ID 9746612), HELO_1644 (NCBI-gene ID 9745660) and the fourth one (154 amino acid residues) was HELO_3812 (NCBI-gene ID 9746788). CLUSTAL 2.1 multiple sequence alignment as well as rooted phylogenetic tree (neighbor joining) were generated for both 12 kDa and 8 kDa proteins. These results, presented as supplementary material, indicated that 12 kDa protein and 8 kDa proteins are related to HELO_3812 and HELO_1644 respectively (supporting information, File S3). Hence, the presence of conserved cold shock domains establishes the identity of 12 kDa and 8 kDa proteins as cold shock proteins without any uncertainty. Thus, in contrast to the induction of major cold shock proteins up on cold shock treatment in other organisms, we report the presence of cold shock proteins in abundance in the absence of any cold shock in H. eurihalina. We also report for the first time continued presence of a cold shock protein (12 kDa) in both exponential and stationary phases of growth. However, 8 kDa cold shock protein is abundant in exponential phase and is almost undetectable in stationary phase of growth similar to CspA which is present in very large amount (up to 2% of the cellular protein) in early exponential phase and decreases drastically during stationary phase as reported by Brandi et al. [6]. Furthermore, both 12 kDa and 8 kDa proteins are strong polypyrimidine binding proteins. In this respect, these proteins are like CspB of *Bacillus subtilis* and TmCsp (Csp from *Thermotoga maritima*), the CSPs which are induced during cold shock, in binding to single stranded DNA and single stranded RNA containing polypyrimidine regions [12]. It has also been reported that CspB and CspC are major stationary phase induced proteins in *B. subtilis* indicating CSPs are inducible during other stress conditions [23]. Our present findings suggest that cold shock proteins are expressed without cold shock under salt stress conditions and that they may have a function in normal cellular metabolism in moderate halophiles. Presence of cold shock proteins in *H. eurihalina*, which is exposed to continuous salt stress by virtue of its halophilic nature, may be contributing as an adaptive mechanism for the salt stress probably destabilizing secondary structures in RNAs.

**Table 1:** Identification of *H. eurihalina* poly (U) binding proteins by MALDI-TOF MS analysis.

Spot no	Precursor ion mass (M+H)^+^	Peptide sequences matched	Observed Mr. on gel	Theoretical Mr.	Protein ID	Accession no.	Reference organism	MS/MS score
1	2757.88	KSLEEGQAVEFEVVEGDRGPQAANVVK.L	12 kDa	7.3 kDa	Major cold shock protein	gi/15924392	*staphylococcus aureus*	104
2	1863.84	KTLAEGQKVEFTVTQGQKG	8 kDa	7.2 kDa	CSD domain family protein	gi/88800616	*Reineka sp.* MED 297	78

### Conclusions

Two cold shock proteins (12 kDa and 8 kDa) are present in *H. eurihalina*, without cold shock treatment. 12 kDa cold shock protein is present throughout the growth cycle from exponential to stationary phase. 8 kDa cold shock protein is not present in stationary phase. Cold shock proteins are purified in a single step by affinity chromatography on Poly (U) Cellulose. Poly (U) cellulose chromatography can be used for rapid purification CSPs with OB fold structure. Bioinformatic analysis suggests that 12 kDa protein and 8 kDa protein are related to HELO_3812 and HELO_1644 of *Halomonas elongata* respectively.

## Supporting Information

File S1 Peptide Mass Fingerprinting and MASCOT Search results of 12 kDa protein.
(DOC)

File S2 Peptide Mass Fingerprinting and MASCOT Search results of 8 kDa protein.
(DOC)

File S3 Bioinformatic analysis for finding homologues of 12 kDa and 8 kDa cold shock proteins of *Halomonas eurihalina*.
(DOC)

## References

[1] Graumann PL, Marahiel MA (1998). superfamily of proteins that contain the cold-shock domain.. Trends Biochem Sci.

[2] Graumann PL, Marahiel MA (1999). Cold shock response in *Bacillus subtilis*.. J Mol Microbiol Biotechnol.

[3] Thieringer HA, Jones PG, Inouye M (1998). Cold shock and adaptation.. Bioassays.

[4] Phadtare S, Alsina J, Inouye M (1999). Cold-shock response and cold-shock proteins.. Curr Opinion Microbiol.

[5] Etchegaray JP, Inouye M (1999). CspA, CspB and CspG, major cold shock proteins of *Escherichia coli* are induced at low temperature under conditions that completely block protein synthesis.. J Bacteriol.

[6] Brandi A, Spurio R, Gualerzil CO, Pon CL (1999). Massive presence of the Escherichia coli ‘major cold-shock protein’ CspA under non-stress conditions.. EMBO J.

[7] Jiang W, Hou Y, Inouye M (1997). CspA, the major cold-shock protein of *Escherichia coli*, is an RNA chaperone.. J Biol Chem.

[8] Bae W, Xia B, Inouye M, Severinov K (2000). *Escherichia coli* CspA-family RNA chaperones are transcription antiterminators.. Proc Natl Acad Sci USA.

[9] Phadtare S, Zhu L, Uemori T, Mukai, Kato I (2009). Applications of nucleic acid chaperone activity of CspA and its homologues.. J Mol Microbiol Biotechnol.

[10] Giaquinto L, Curmi PMG, Siddiqui KS, Poljak A, DeLong E (2007). Structure and Function of Cold Shock Proteins in Archaea.. J Bacteriol.

[11] Ermolenkoa DN, Makhatadze GI (2002). Bacterial cold-shock proteins.. Cell Mol Life Sci.

[12] Horn G, Hofweber R, Kremer W, Kalbitzer HR (2007). Structure and function of bacterial cold shock proteins.. Cell Mol Life Sci.

[13] Phadtare S, Severinov K (2010). RNA remodeling and gene regulation by cold shock proteins.. RNA Biology.

[14] Carmichael CG (1975). Isolation of bacterial and phage proteins by homopolymer RNA cellulose chromatography.. J Biol Chem.

[15] Suryanarayana T, Subramanian AR (1983). An essential function of ribosomal protein S1 in messenger ribonucleic acid translation.. Biochemistry.

[16] Minks MA, Suryanarayana T, Subramanian AR (1978). Metabolic stability of the two forms of initiation factor IF-3 in *Escherichia coli* during the growth cycle.. Eur J Biochem.

[17] Thomas JO, Kornberg RD (1975). An octamer of histones in chromatin and free in solution.. Proc Natl Acad Sci USA.

[18] Madjar JJ, Michel S, Cozzone AJ, Reboud JP (1979). A method to identify individual proteins in four different two dimensional gel electrophoresis systems: Application to *E. coli* ribosomal proteins.. Anal Biochem.

[19] Metz LJ, Bogorad L (1974). Two-dimensional polyacrylamide gel electrophoresis: an improved method for ribosomal proteins.. Anal Biochem.

[20] Laemmli UK (1970). Cleavage of structural proteins during the assembly of the head of bacteriophage T4.. Nature.

[21] Shevchenko A, Wilm A, Vorm O, Mann M (1996). Mass spectrometric sequencing of protein from silver-stained polyacrylamide gels,. Anal Chem.

[22] Wolffe AP, Tafuri S, Ranjan M, Familari M (1992). The Y-box factors: a family of nucleic acid binding proteins conserved from *Escherichia coli* to man.. New Biol.

[23] Graumann PL, Marahiel MA (1999). Cold shock proteins CspB and CspC are major stationary-phase-induced proteins in Bacillus subtilis.. Arch Microbiol.

