# Supplementary material for: Isolation of Two Strong Poly (U) Binding Proteins from Moderate Halophile Halomonas eurihalina and Their Identification as Cold Shock Proteins
Source: PLoS One. 2012 Apr 13;7(4):e34409. doi: 10.1371/journal.pone.0034409 (PMC3326018; doi:10.1371/journal.pone.0034409)
Supplement: File S1 — Peptide Mass Fingerprinting and MASCOT Search results of 12 kDa protein. (DOC) [file pone.0034409.s001.doc]

File S1

Peptide Mass Fingerprinting and MASCOT Search results of 12 kDa protein (spot No.1)

**
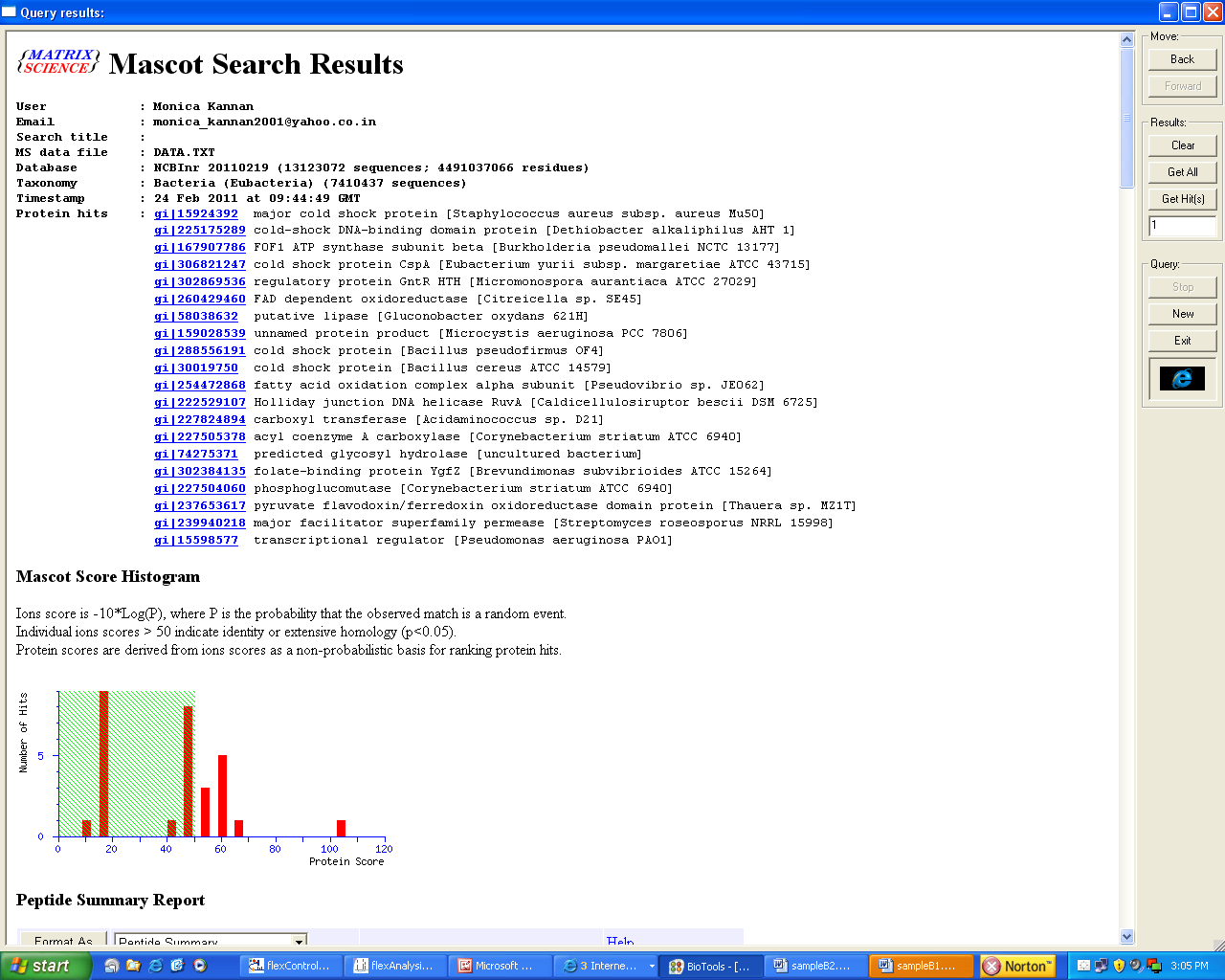
**

| **1.** | [gi|15924392](http://www.matrixscience.com/cgi/protein_view.pl?file=../data/20110224/FttoIeSte.dat&hit=gi|15924392&db_idx=1&px=1&ave_thresh=50&_ignoreionsscorebelow=0&report=20&_sigthreshold=0.05&_msresflags=1025&_msresflags2=2&percolate=-1&percolate_rt=0)    **Mass:** 7317     **Score:** 104    **Matches:** 1(1)  **Sequences:** 1(1) |
| --- | --- |
|  | major cold shock protein [Staphylococcus aureus subsp. aureus Mu50] |

|  | Check to include this hit in error tolerant search |
| --- | --- |
|  |  |

|  |  | **Query** | | **Observed** | **Mr(expt)** | **Mr(calc)** | **ppm** | **Miss** | **Score** | **Expect** | **Rank** | | **Unique** | **Peptide** |
| --- | --- | --- | --- | --- | --- | --- | --- | --- | --- | --- | --- | --- | --- | --- |
|  |  | [7](http://www.matrixscience.com/cgi/peptide_view.pl?file=../data/20110224/FttoIeSte.dat&query=7&hit=1&index=gi|15924392&px=1&section=5&ave_thresh=50&_ignoreionsscorebelow=0&report=20&_sigthreshold=0.05&_msresflags=1025&_msresflags2=2&percolate=-1&percolate_rt=0) | | **2757.8882** | **2756.8809** | **2756.3617** | **188** | **1** | **104** | **2.3e-07** | **1** | | **U** | **K.SLEEGQAVEFEVVEGDRGPQAANVVK.L** |
|  | | | | | | | | | | | |  | | |
|  | | | **Proteins matching the same set of peptides:** | | | | | | | | |  | | |

|  | [gi|20302392](http://www.matrixscience.com/cgi/protein_view.pl?file=../data/20110224/FttoIeSte.dat&hit=gi|20302392&db_idx=1&px=1&ave_thresh=50&_ignoreionsscorebelow=0&report=20&_sigthreshold=0.05&_msresflags=1025&_msresflags2=2&percolate=-1&percolate_rt=0)    **Mass:** 7283     **Score:** 104    **Matches:** 1(1)  **Sequences:** 1(1) |
| --- | --- |
|  | cold shock protein [Staphylococcus aureus] |

|  | [gi|224476532](http://www.matrixscience.com/cgi/protein_view.pl?file=../data/20110224/FttoIeSte.dat&hit=gi|224476532&db_idx=1&px=1&ave_thresh=50&_ignoreionsscorebelow=0&report=20&_sigthreshold=0.05&_msresflags=1025&_msresflags2=2&percolate=-1&percolate_rt=0)    **Mass:** 7331     **Score:** 104    **Matches:** 1(1)  **Sequences:** 1(1) |
| --- | --- |
|  | putative cold shock protein [Staphylococcus carnosus subsp. carnosus TM300] |

| **2.** | [gi|225175289](http://www.matrixscience.com/cgi/protein_view.pl?file=../data/20110224/FttoIeSte.dat&hit=gi|225175289&db_idx=1&px=1&ave_thresh=50&_ignoreionsscorebelow=0&report=20&_sigthreshold=0.05&_msresflags=1025&_msresflags2=2&percolate=-1&percolate_rt=0)    **Mass:** 7289     **Score:** 65     **Matches:** 1(1)  **Sequences:** 1(1) |
| --- | --- |
|  | cold-shock DNA-binding domain protein [Dethiobacter alkaliphilus AHT 1] |

|  | Check to include this hit in error tolerant search |
| --- | --- |
|  |  |

|  | **Query** | **Observed** | **Mr(expt)** | **Mr(calc)** | **ppm** | **Miss** | **Score** | **Expect** | **Rank** | **Unique** | **Peptide** |
| --- | --- | --- | --- | --- | --- | --- | --- | --- | --- | --- | --- |
|  | [7](http://www.matrixscience.com/cgi/peptide_view.pl?file=../data/20110224/FttoIeSte.dat&query=7&hit=2&index=gi|225175289&px=1&section=5&ave_thresh=50&_ignoreionsscorebelow=0&report=20&_sigthreshold=0.05&_msresflags=1025&_msresflags2=2&percolate=-1&percolate_rt=0) | 2757.8882 | 2756.8809 | 2757.3821 | -181.75 | 1 | 65 | 0.0019 | 2 | U | K.SLAEGEEVEFEIVEGTRGPQAANVVK.L |

Top of Form

Match to: **gi|15924392** Score: **104**

**major cold shock protein [Staphylococcus aureus subsp. aureus Mu50]**

Found in search of DATA.TXT

Nominal mass (M_r_): **7317**; Calculated pI value: **4.52**

NCBI BLAST search of [gi|15924392](http://www.ncbi.nlm.nih.gov/blast/Blast.cgi?ALIGNMENTS=50&ALIGNMENT_VIEW=Pairwise&AUTO_FORMAT=Semiauto&CDD_SEARCH=on&CLIENT=web&COMPOSITION_BASED_STATISTICS=on&DATABASE=nr&DESCRIPTIONS=100&ENTREZ_QUERY=(none)&EXPECT=10&FILTER=L&FORMAT_BLOCK_ON_RESPAGE=None&FORMAT_OBJECT=Alignment&FORMAT_TYPE=HTML&GAPCOSTS=11+1&I_THRESH=0.001&LAYOUT=TwoWindows&MATRIX_NAME=BLOSUM62&NCBI_GI=on&PAGE=Proteins&PROGRAM=blastp&QUERY=MKQGTVKWFNAEKGFGFIEVEGENDVFVHFSAINQDGYKSLEEGQAVEFEVVEGDRGPQAANVVKL&SERVICE=plain&SET_DEFAULTS.x=9&SET_DEFAULTS.y=5&SHOW_OVERVIEW=on&WORD_SIZE=3&END_OF_HTTPGET=Yes) against nr

Unformatted [sequence string](http://www.matrixscience.com/cgi/getseq.pl?NCBInr+gi|15924392+seq) for pasting into other applications

Taxonomy: [Staphylococcus aureus subsp. aureus Mu50](http://www.ncbi.nlm.nih.gov/Taxonomy/Browser/wwwtax.cgi?lvl=0&id=158878)

Links to retrieve other entries containing this sequence from NCBI Entrez:

[gi|15926982](http://www.ncbi.nlm.nih.gov/entrez/query.fcgi?cmd=search&db=protein&doptcmdl=genpept&tool=mascot&term=15926982) from [Staphylococcus aureus subsp. aureus N315](http://www.ncbi.nlm.nih.gov/Taxonomy/Browser/wwwtax.cgi?lvl=0&id=158879)

[gi|21283019](http://www.ncbi.nlm.nih.gov/entrez/query.fcgi?cmd=search&db=protein&doptcmdl=genpept&tool=mascot&term=21283019) from [Staphylococcus aureus subsp. aureus MW2](http://www.ncbi.nlm.nih.gov/Taxonomy/Browser/wwwtax.cgi?lvl=0&id=196620)

[gi|49483592](http://www.ncbi.nlm.nih.gov/entrez/query.fcgi?cmd=search&db=protein&doptcmdl=genpept&tool=mascot&term=49483592) from [Staphylococcus aureus subsp. aureus MRSA252](http://www.ncbi.nlm.nih.gov/Taxonomy/Browser/wwwtax.cgi?lvl=0&id=282458)

[gi|49486243](http://www.ncbi.nlm.nih.gov/entrez/query.fcgi?cmd=search&db=protein&doptcmdl=genpept&tool=mascot&term=49486243) from [Staphylococcus aureus subsp. aureus MSSA476](http://www.ncbi.nlm.nih.gov/Taxonomy/Browser/wwwtax.cgi?lvl=0&id=282459)

[gi|57651902](http://www.ncbi.nlm.nih.gov/entrez/query.fcgi?cmd=search&db=protein&doptcmdl=genpept&tool=mascot&term=57651902) from [Staphylococcus aureus subsp. aureus COL](http://www.ncbi.nlm.nih.gov/Taxonomy/Browser/wwwtax.cgi?lvl=0&id=93062)

[gi|82750994](http://www.ncbi.nlm.nih.gov/entrez/query.fcgi?cmd=search&db=protein&doptcmdl=genpept&tool=mascot&term=82750994) from [Staphylococcus aureus RF122](http://www.ncbi.nlm.nih.gov/Taxonomy/Browser/wwwtax.cgi?lvl=0&id=273036)

[gi|87161577](http://www.ncbi.nlm.nih.gov/entrez/query.fcgi?cmd=search&db=protein&doptcmdl=genpept&tool=mascot&term=87161577) from [Staphylococcus aureus subsp. aureus USA300_FPR3757](http://www.ncbi.nlm.nih.gov/Taxonomy/Browser/wwwtax.cgi?lvl=0&id=451515)

[gi|88195129](http://www.ncbi.nlm.nih.gov/entrez/query.fcgi?cmd=search&db=protein&doptcmdl=genpept&tool=mascot&term=88195129) from [Staphylococcus aureus subsp. aureus NCTC 8325](http://www.ncbi.nlm.nih.gov/Taxonomy/Browser/wwwtax.cgi?lvl=0&id=93061)

[gi|148267890](http://www.ncbi.nlm.nih.gov/entrez/query.fcgi?cmd=search&db=protein&doptcmdl=genpept&tool=mascot&term=148267890) from [Staphylococcus aureus subsp. aureus JH9](http://www.ncbi.nlm.nih.gov/Taxonomy/Browser/wwwtax.cgi?lvl=0&id=359786)

[gi|150393953](http://www.ncbi.nlm.nih.gov/entrez/query.fcgi?cmd=search&db=protein&doptcmdl=genpept&tool=mascot&term=150393953) from [Staphylococcus aureus subsp. aureus JH1](http://www.ncbi.nlm.nih.gov/Taxonomy/Browser/wwwtax.cgi?lvl=0&id=359787)

[gi|151221525](http://www.ncbi.nlm.nih.gov/entrez/query.fcgi?cmd=search&db=protein&doptcmdl=genpept&tool=mascot&term=151221525) from [Staphylococcus aureus subsp. aureus str. Newman](http://www.ncbi.nlm.nih.gov/Taxonomy/Browser/wwwtax.cgi?lvl=0&id=426430)

[gi|156979721](http://www.ncbi.nlm.nih.gov/entrez/query.fcgi?cmd=search&db=protein&doptcmdl=genpept&tool=mascot&term=156979721) from [Staphylococcus aureus subsp. aureus Mu3](http://www.ncbi.nlm.nih.gov/Taxonomy/Browser/wwwtax.cgi?lvl=0&id=418127)

[gi|161509568](http://www.ncbi.nlm.nih.gov/entrez/query.fcgi?cmd=search&db=protein&doptcmdl=genpept&tool=mascot&term=161509568) from [Staphylococcus aureus subsp. aureus USA300_TCH1516](http://www.ncbi.nlm.nih.gov/Taxonomy/Browser/wwwtax.cgi?lvl=0&id=451516)

[gi|221140629](http://www.ncbi.nlm.nih.gov/entrez/query.fcgi?cmd=search&db=protein&doptcmdl=genpept&tool=mascot&term=221140629) from [Staphylococcus aureus subsp. aureus str. JKD6009](http://www.ncbi.nlm.nih.gov/Taxonomy/Browser/wwwtax.cgi?lvl=0&id=546343)

[gi|253315273](http://www.ncbi.nlm.nih.gov/entrez/query.fcgi?cmd=search&db=protein&doptcmdl=genpept&tool=mascot&term=253315273) from [Staphylococcus aureus subsp. aureus str. CF-Marseille](http://www.ncbi.nlm.nih.gov/Taxonomy/Browser/wwwtax.cgi?lvl=0&id=505321)

[gi|253732039](http://www.ncbi.nlm.nih.gov/entrez/query.fcgi?cmd=search&db=protein&doptcmdl=genpept&tool=mascot&term=253732039) from [Staphylococcus aureus subsp. aureus USA300_TCH959](http://www.ncbi.nlm.nih.gov/Taxonomy/Browser/wwwtax.cgi?lvl=0&id=450394)

[gi|253733351](http://www.ncbi.nlm.nih.gov/entrez/query.fcgi?cmd=search&db=protein&doptcmdl=genpept&tool=mascot&term=253733351) from [Staphylococcus aureus subsp. aureus TCH130](http://www.ncbi.nlm.nih.gov/Taxonomy/Browser/wwwtax.cgi?lvl=0&id=548474)

[gi|255006191](http://www.ncbi.nlm.nih.gov/entrez/query.fcgi?cmd=search&db=protein&doptcmdl=genpept&tool=mascot&term=255006191) from [Staphylococcus aureus subsp. aureus Mu50-omega](http://www.ncbi.nlm.nih.gov/Taxonomy/Browser/wwwtax.cgi?lvl=0&id=585891)

[gi|257425466](http://www.ncbi.nlm.nih.gov/entrez/query.fcgi?cmd=search&db=protein&doptcmdl=genpept&tool=mascot&term=257425466) from [Staphylococcus aureus subsp. aureus 55/2053](http://www.ncbi.nlm.nih.gov/Taxonomy/Browser/wwwtax.cgi?lvl=0&id=585143)

[gi|257428126](http://www.ncbi.nlm.nih.gov/entrez/query.fcgi?cmd=search&db=protein&doptcmdl=genpept&tool=mascot&term=257428126) from [Staphylococcus aureus subsp. aureus 65-1322](http://www.ncbi.nlm.nih.gov/Taxonomy/Browser/wwwtax.cgi?lvl=0&id=585145)

[gi|257430757](http://www.ncbi.nlm.nih.gov/entrez/query.fcgi?cmd=search&db=protein&doptcmdl=genpept&tool=mascot&term=257430757) from [Staphylococcus aureus subsp. aureus 68-397](http://www.ncbi.nlm.nih.gov/Taxonomy/Browser/wwwtax.cgi?lvl=0&id=585146)

[gi|257433517](http://www.ncbi.nlm.nih.gov/entrez/query.fcgi?cmd=search&db=protein&doptcmdl=genpept&tool=mascot&term=257433517) from [Staphylococcus aureus subsp. aureus E1410](http://www.ncbi.nlm.nih.gov/Taxonomy/Browser/wwwtax.cgi?lvl=0&id=585153)

[gi|257436358](http://www.ncbi.nlm.nih.gov/entrez/query.fcgi?cmd=search&db=protein&doptcmdl=genpept&tool=mascot&term=257436358) from [Staphylococcus aureus subsp. aureus M876](http://www.ncbi.nlm.nih.gov/Taxonomy/Browser/wwwtax.cgi?lvl=0&id=585158)

[gi|257795543](http://www.ncbi.nlm.nih.gov/entrez/query.fcgi?cmd=search&db=protein&doptcmdl=genpept&tool=mascot&term=257795543) from [Staphylococcus aureus A9781](http://www.ncbi.nlm.nih.gov/Taxonomy/Browser/wwwtax.cgi?lvl=0&id=553596)

[gi|258413351](http://www.ncbi.nlm.nih.gov/entrez/query.fcgi?cmd=search&db=protein&doptcmdl=genpept&tool=mascot&term=258413351) from [Staphylococcus aureus A9763](http://www.ncbi.nlm.nih.gov/Taxonomy/Browser/wwwtax.cgi?lvl=0&id=553592)

[gi|258420542](http://www.ncbi.nlm.nih.gov/entrez/query.fcgi?cmd=search&db=protein&doptcmdl=genpept&tool=mascot&term=258420542) from [Staphylococcus aureus A9719](http://www.ncbi.nlm.nih.gov/Taxonomy/Browser/wwwtax.cgi?lvl=0&id=553588)

[gi|258423666](http://www.ncbi.nlm.nih.gov/entrez/query.fcgi?cmd=search&db=protein&doptcmdl=genpept&tool=mascot&term=258423666) from [Staphylococcus aureus A9635](http://www.ncbi.nlm.nih.gov/Taxonomy/Browser/wwwtax.cgi?lvl=0&id=553583)

[gi|258434686](http://www.ncbi.nlm.nih.gov/entrez/query.fcgi?cmd=search&db=protein&doptcmdl=genpept&tool=mascot&term=258434686) from [Staphylococcus aureus A9299](http://www.ncbi.nlm.nih.gov/Taxonomy/Browser/wwwtax.cgi?lvl=0&id=553581)

[gi|258444738](http://www.ncbi.nlm.nih.gov/entrez/query.fcgi?cmd=search&db=protein&doptcmdl=genpept&tool=mascot&term=258444738) from [Staphylococcus aureus A8115](http://www.ncbi.nlm.nih.gov/Taxonomy/Browser/wwwtax.cgi?lvl=0&id=553573)

[gi|258447427](http://www.ncbi.nlm.nih.gov/entrez/query.fcgi?cmd=search&db=protein&doptcmdl=genpept&tool=mascot&term=258447427) from [Staphylococcus aureus A6300](http://www.ncbi.nlm.nih.gov/Taxonomy/Browser/wwwtax.cgi?lvl=0&id=553571)

[gi|258449268](http://www.ncbi.nlm.nih.gov/entrez/query.fcgi?cmd=search&db=protein&doptcmdl=genpept&tool=mascot&term=258449268) from [Staphylococcus aureus A6224](http://www.ncbi.nlm.nih.gov/Taxonomy/Browser/wwwtax.cgi?lvl=0&id=553568)

[gi|258452191](http://www.ncbi.nlm.nih.gov/entrez/query.fcgi?cmd=search&db=protein&doptcmdl=genpept&tool=mascot&term=258452191) from [Staphylococcus aureus A5948](http://www.ncbi.nlm.nih.gov/Taxonomy/Browser/wwwtax.cgi?lvl=0&id=553567)

[gi|258454648](http://www.ncbi.nlm.nih.gov/entrez/query.fcgi?cmd=search&db=protein&doptcmdl=genpept&tool=mascot&term=258454648) from [Staphylococcus aureus A5937](http://www.ncbi.nlm.nih.gov/Taxonomy/Browser/wwwtax.cgi?lvl=0&id=553565)

[gi|262050357](http://www.ncbi.nlm.nih.gov/entrez/query.fcgi?cmd=search&db=protein&doptcmdl=genpept&tool=mascot&term=262050357) from [Staphylococcus aureus D30](http://www.ncbi.nlm.nih.gov/Taxonomy/Browser/wwwtax.cgi?lvl=0&id=455227)

[gi|269203025](http://www.ncbi.nlm.nih.gov/entrez/query.fcgi?cmd=search&db=protein&doptcmdl=genpept&tool=mascot&term=269203025) from [Staphylococcus aureus subsp. aureus ED98](http://www.ncbi.nlm.nih.gov/Taxonomy/Browser/wwwtax.cgi?lvl=0&id=681288)

[gi|282892895](http://www.ncbi.nlm.nih.gov/entrez/query.fcgi?cmd=search&db=protein&doptcmdl=genpept&tool=mascot&term=282892895) from [Staphylococcus aureus A8117](http://www.ncbi.nlm.nih.gov/Taxonomy/Browser/wwwtax.cgi?lvl=0&id=553574)

[gi|282903982](http://www.ncbi.nlm.nih.gov/entrez/query.fcgi?cmd=search&db=protein&doptcmdl=genpept&tool=mascot&term=282903982) from [Staphylococcus aureus subsp. aureus C160](http://www.ncbi.nlm.nih.gov/Taxonomy/Browser/wwwtax.cgi?lvl=0&id=585150)

[gi|282905747](http://www.ncbi.nlm.nih.gov/entrez/query.fcgi?cmd=search&db=protein&doptcmdl=genpept&tool=mascot&term=282905747) from [Staphylococcus aureus subsp. aureus Btn1260](http://www.ncbi.nlm.nih.gov/Taxonomy/Browser/wwwtax.cgi?lvl=0&id=585148)

[gi|282908718](http://www.ncbi.nlm.nih.gov/entrez/query.fcgi?cmd=search&db=protein&doptcmdl=genpept&tool=mascot&term=282908718) from [Staphylococcus aureus subsp. aureus WW2703/97](http://www.ncbi.nlm.nih.gov/Taxonomy/Browser/wwwtax.cgi?lvl=0&id=585161)

[gi|282910982](http://www.ncbi.nlm.nih.gov/entrez/query.fcgi?cmd=search&db=protein&doptcmdl=genpept&tool=mascot&term=282910982) from [Staphylococcus aureus subsp. aureus WBG10049](http://www.ncbi.nlm.nih.gov/Taxonomy/Browser/wwwtax.cgi?lvl=0&id=585160)

[gi|282914190](http://www.ncbi.nlm.nih.gov/entrez/query.fcgi?cmd=search&db=protein&doptcmdl=genpept&tool=mascot&term=282914190) from [Staphylococcus aureus subsp. aureus M899](http://www.ncbi.nlm.nih.gov/Taxonomy/Browser/wwwtax.cgi?lvl=0&id=585159)

[gi|282916668](http://www.ncbi.nlm.nih.gov/entrez/query.fcgi?cmd=search&db=protein&doptcmdl=genpept&tool=mascot&term=282916668) from [Staphylococcus aureus subsp. aureus D139](http://www.ncbi.nlm.nih.gov/Taxonomy/Browser/wwwtax.cgi?lvl=0&id=585152)

[gi|282919112](http://www.ncbi.nlm.nih.gov/entrez/query.fcgi?cmd=search&db=protein&doptcmdl=genpept&tool=mascot&term=282919112) from [Staphylococcus aureus subsp. aureus C427](http://www.ncbi.nlm.nih.gov/Taxonomy/Browser/wwwtax.cgi?lvl=0&id=585151)

[gi|282924295](http://www.ncbi.nlm.nih.gov/entrez/query.fcgi?cmd=search&db=protein&doptcmdl=genpept&tool=mascot&term=282924295) from [Staphylococcus aureus subsp. aureus C101](http://www.ncbi.nlm.nih.gov/Taxonomy/Browser/wwwtax.cgi?lvl=0&id=585149)

[gi|282927926](http://www.ncbi.nlm.nih.gov/entrez/query.fcgi?cmd=search&db=protein&doptcmdl=genpept&tool=mascot&term=282927926) from [Staphylococcus aureus A10102](http://www.ncbi.nlm.nih.gov/Taxonomy/Browser/wwwtax.cgi?lvl=0&id=553601)

[gi|282929069](http://www.ncbi.nlm.nih.gov/entrez/query.fcgi?cmd=search&db=protein&doptcmdl=genpept&tool=mascot&term=282929069) from [Staphylococcus aureus A9765](http://www.ncbi.nlm.nih.gov/Taxonomy/Browser/wwwtax.cgi?lvl=0&id=553594)

[gi|283770473](http://www.ncbi.nlm.nih.gov/entrez/query.fcgi?cmd=search&db=protein&doptcmdl=genpept&tool=mascot&term=283770473) from [Staphylococcus aureus subsp. aureus H19](http://www.ncbi.nlm.nih.gov/Taxonomy/Browser/wwwtax.cgi?lvl=0&id=585155)

[gi|283958164](http://www.ncbi.nlm.nih.gov/entrez/query.fcgi?cmd=search&db=protein&doptcmdl=genpept&tool=mascot&term=283958164) from [Staphylococcus aureus subsp. aureus A017934/97](http://www.ncbi.nlm.nih.gov/Taxonomy/Browser/wwwtax.cgi?lvl=0&id=585147)

[gi|284024402](http://www.ncbi.nlm.nih.gov/entrez/query.fcgi?cmd=search&db=protein&doptcmdl=genpept&tool=mascot&term=284024402) from [Staphylococcus aureus subsp. aureus 132](http://www.ncbi.nlm.nih.gov/Taxonomy/Browser/wwwtax.cgi?lvl=0&id=644279)

[gi|293501217](http://www.ncbi.nlm.nih.gov/entrez/query.fcgi?cmd=search&db=protein&doptcmdl=genpept&tool=mascot&term=293501217) from [Staphylococcus aureus subsp. aureus 58-424](http://www.ncbi.nlm.nih.gov/Taxonomy/Browser/wwwtax.cgi?lvl=0&id=585144)

[gi|293510178](http://www.ncbi.nlm.nih.gov/entrez/query.fcgi?cmd=search&db=protein&doptcmdl=genpept&tool=mascot&term=293510178) from [Staphylococcus aureus subsp. aureus M809](http://www.ncbi.nlm.nih.gov/Taxonomy/Browser/wwwtax.cgi?lvl=0&id=585157)

[gi|293526770](http://www.ncbi.nlm.nih.gov/entrez/query.fcgi?cmd=search&db=protein&doptcmdl=genpept&tool=mascot&term=293526770) from [Staphylococcus aureus subsp. aureus M1015](http://www.ncbi.nlm.nih.gov/Taxonomy/Browser/wwwtax.cgi?lvl=0&id=585156)

[gi|294850737](http://www.ncbi.nlm.nih.gov/entrez/query.fcgi?cmd=search&db=protein&doptcmdl=genpept&tool=mascot&term=294850737) from [Staphylococcus aureus A9754](http://www.ncbi.nlm.nih.gov/Taxonomy/Browser/wwwtax.cgi?lvl=0&id=553590)

[gi|295406349](http://www.ncbi.nlm.nih.gov/entrez/query.fcgi?cmd=search&db=protein&doptcmdl=genpept&tool=mascot&term=295406349) from [Staphylococcus aureus A8819](http://www.ncbi.nlm.nih.gov/Taxonomy/Browser/wwwtax.cgi?lvl=0&id=553580)

[gi|295427915](http://www.ncbi.nlm.nih.gov/entrez/query.fcgi?cmd=search&db=protein&doptcmdl=genpept&tool=mascot&term=295427915) from [Staphylococcus aureus subsp. aureus EMRSA16](http://www.ncbi.nlm.nih.gov/Taxonomy/Browser/wwwtax.cgi?lvl=0&id=585154)

[gi|296275262](http://www.ncbi.nlm.nih.gov/entrez/query.fcgi?cmd=search&db=protein&doptcmdl=genpept&tool=mascot&term=296275262) from [Staphylococcus aureus subsp. aureus MR1](http://www.ncbi.nlm.nih.gov/Taxonomy/Browser/wwwtax.cgi?lvl=0&id=680649)

[gi|297207943](http://www.ncbi.nlm.nih.gov/entrez/query.fcgi?cmd=search&db=protein&doptcmdl=genpept&tool=mascot&term=297207943) from [Staphylococcus aureus subsp. aureus ATCC 51811](http://www.ncbi.nlm.nih.gov/Taxonomy/Browser/wwwtax.cgi?lvl=0&id=762962)

[gi|297244578](http://www.ncbi.nlm.nih.gov/entrez/query.fcgi?cmd=search&db=protein&doptcmdl=genpept&tool=mascot&term=297244578) from [Staphylococcus aureus A8796](http://www.ncbi.nlm.nih.gov/Taxonomy/Browser/wwwtax.cgi?lvl=0&id=553577)

[gi|297591124](http://www.ncbi.nlm.nih.gov/entrez/query.fcgi?cmd=search&db=protein&doptcmdl=genpept&tool=mascot&term=297591124) from [Staphylococcus aureus subsp. aureus MN8](http://www.ncbi.nlm.nih.gov/Taxonomy/Browser/wwwtax.cgi?lvl=0&id=548470)

[gi|300912027](http://www.ncbi.nlm.nih.gov/entrez/query.fcgi?cmd=search&db=protein&doptcmdl=genpept&tool=mascot&term=300912027) from [Staphylococcus aureus subsp. aureus TCH70](http://www.ncbi.nlm.nih.gov/Taxonomy/Browser/wwwtax.cgi?lvl=0&id=548475)

[gi|304381021](http://www.ncbi.nlm.nih.gov/entrez/query.fcgi?cmd=search&db=protein&doptcmdl=genpept&tool=mascot&term=304381021) from [Staphylococcus aureus subsp. aureus ATCC BAA-39](http://www.ncbi.nlm.nih.gov/Taxonomy/Browser/wwwtax.cgi?lvl=0&id=862516)

[gi|81827857](http://www.ncbi.nlm.nih.gov/entrez/query.fcgi?cmd=search&db=protein&doptcmdl=genpept&tool=mascot&term=81827857) from [Staphylococcus aureus subsp. aureus MSSA476](http://www.ncbi.nlm.nih.gov/Taxonomy/Browser/wwwtax.cgi?lvl=0&id=282459)

[gi|81828019](http://www.ncbi.nlm.nih.gov/entrez/query.fcgi?cmd=search&db=protein&doptcmdl=genpept&tool=mascot&term=81828019) from [Staphylococcus aureus subsp. aureus MRSA252](http://www.ncbi.nlm.nih.gov/Taxonomy/Browser/wwwtax.cgi?lvl=0&id=282458)

[gi|81832398](http://www.ncbi.nlm.nih.gov/entrez/query.fcgi?cmd=search&db=protein&doptcmdl=genpept&tool=mascot&term=81832398) from [Staphylococcus aureus subsp. aureus MW2](http://www.ncbi.nlm.nih.gov/Taxonomy/Browser/wwwtax.cgi?lvl=0&id=196620)

[gi|81832432](http://www.ncbi.nlm.nih.gov/entrez/query.fcgi?cmd=search&db=protein&doptcmdl=genpept&tool=mascot&term=81832432) from [Staphylococcus aureus subsp. aureus Mu50](http://www.ncbi.nlm.nih.gov/Taxonomy/Browser/wwwtax.cgi?lvl=0&id=158878)

[gi|81832507](http://www.ncbi.nlm.nih.gov/entrez/query.fcgi?cmd=search&db=protein&doptcmdl=genpept&tool=mascot&term=81832507) from [Staphylococcus aureus subsp. aureus N315](http://www.ncbi.nlm.nih.gov/Taxonomy/Browser/wwwtax.cgi?lvl=0&id=158879)

[gi|81859588](http://www.ncbi.nlm.nih.gov/entrez/query.fcgi?cmd=search&db=protein&doptcmdl=genpept&tool=mascot&term=81859588) from [Staphylococcus aureus subsp. aureus COL](http://www.ncbi.nlm.nih.gov/Taxonomy/Browser/wwwtax.cgi?lvl=0&id=93062)

[gi|118572254](http://www.ncbi.nlm.nih.gov/entrez/query.fcgi?cmd=search&db=protein&doptcmdl=genpept&tool=mascot&term=118572254) from [Staphylococcus aureus subsp. aureus USA300](http://www.ncbi.nlm.nih.gov/Taxonomy/Browser/wwwtax.cgi?lvl=0&id=367830)

[gi|118572255](http://www.ncbi.nlm.nih.gov/entrez/query.fcgi?cmd=search&db=protein&doptcmdl=genpept&tool=mascot&term=118572255) from [Staphylococcus aureus subsp. aureus NCTC 8325](http://www.ncbi.nlm.nih.gov/Taxonomy/Browser/wwwtax.cgi?lvl=0&id=93061)

[gi|118572256](http://www.ncbi.nlm.nih.gov/entrez/query.fcgi?cmd=search&db=protein&doptcmdl=genpept&tool=mascot&term=118572256) from [Staphylococcus aureus RF122](http://www.ncbi.nlm.nih.gov/Taxonomy/Browser/wwwtax.cgi?lvl=0&id=273036)

[gi|8101860](http://www.ncbi.nlm.nih.gov/entrez/query.fcgi?cmd=search&db=protein&doptcmdl=genpept&tool=mascot&term=8101860) from [Staphylococcus aureus](http://www.ncbi.nlm.nih.gov/Taxonomy/Browser/wwwtax.cgi?lvl=0&id=1280)

[gi|13701199](http://www.ncbi.nlm.nih.gov/entrez/query.fcgi?cmd=search&db=protein&doptcmdl=genpept&tool=mascot&term=13701199) from [Staphylococcus aureus subsp. aureus N315](http://www.ncbi.nlm.nih.gov/Taxonomy/Browser/wwwtax.cgi?lvl=0&id=158879)

[gi|14247173](http://www.ncbi.nlm.nih.gov/entrez/query.fcgi?cmd=search&db=protein&doptcmdl=genpept&tool=mascot&term=14247173) from [Staphylococcus aureus subsp. aureus Mu50](http://www.ncbi.nlm.nih.gov/Taxonomy/Browser/wwwtax.cgi?lvl=0&id=158878)

[gi|21204458](http://www.ncbi.nlm.nih.gov/entrez/query.fcgi?cmd=search&db=protein&doptcmdl=genpept&tool=mascot&term=21204458) from [Staphylococcus aureus subsp. aureus MW2](http://www.ncbi.nlm.nih.gov/Taxonomy/Browser/wwwtax.cgi?lvl=0&id=196620)

[gi|49241721](http://www.ncbi.nlm.nih.gov/entrez/query.fcgi?cmd=search&db=protein&doptcmdl=genpept&tool=mascot&term=49241721) from [Staphylococcus aureus subsp. aureus MRSA252](http://www.ncbi.nlm.nih.gov/Taxonomy/Browser/wwwtax.cgi?lvl=0&id=282458)

[gi|49244686](http://www.ncbi.nlm.nih.gov/entrez/query.fcgi?cmd=search&db=protein&doptcmdl=genpept&tool=mascot&term=49244686) from [Staphylococcus aureus subsp. aureus MSSA476](http://www.ncbi.nlm.nih.gov/Taxonomy/Browser/wwwtax.cgi?lvl=0&id=282459)

[gi|57286088](http://www.ncbi.nlm.nih.gov/entrez/query.fcgi?cmd=search&db=protein&doptcmdl=genpept&tool=mascot&term=57286088) from [Staphylococcus aureus subsp. aureus COL](http://www.ncbi.nlm.nih.gov/Taxonomy/Browser/wwwtax.cgi?lvl=0&id=93062)

[gi|82656525](http://www.ncbi.nlm.nih.gov/entrez/query.fcgi?cmd=search&db=protein&doptcmdl=genpept&tool=mascot&term=82656525) from [Staphylococcus aureus RF122](http://www.ncbi.nlm.nih.gov/Taxonomy/Browser/wwwtax.cgi?lvl=0&id=273036)

[gi|87127551](http://www.ncbi.nlm.nih.gov/entrez/query.fcgi?cmd=search&db=protein&doptcmdl=genpept&tool=mascot&term=87127551) from [Staphylococcus aureus subsp. aureus USA300_FPR3757](http://www.ncbi.nlm.nih.gov/Taxonomy/Browser/wwwtax.cgi?lvl=0&id=451515)

[gi|87202687](http://www.ncbi.nlm.nih.gov/entrez/query.fcgi?cmd=search&db=protein&doptcmdl=genpept&tool=mascot&term=87202687) from [Staphylococcus aureus subsp. aureus NCTC 8325](http://www.ncbi.nlm.nih.gov/Taxonomy/Browser/wwwtax.cgi?lvl=0&id=93061)

[gi|147740959](http://www.ncbi.nlm.nih.gov/entrez/query.fcgi?cmd=search&db=protein&doptcmdl=genpept&tool=mascot&term=147740959) from [Staphylococcus aureus subsp. aureus JH9](http://www.ncbi.nlm.nih.gov/Taxonomy/Browser/wwwtax.cgi?lvl=0&id=359786)

[gi|149946405](http://www.ncbi.nlm.nih.gov/entrez/query.fcgi?cmd=search&db=protein&doptcmdl=genpept&tool=mascot&term=149946405) from [Staphylococcus aureus subsp. aureus JH1](http://www.ncbi.nlm.nih.gov/Taxonomy/Browser/wwwtax.cgi?lvl=0&id=359787)

[gi|150374325](http://www.ncbi.nlm.nih.gov/entrez/query.fcgi?cmd=search&db=protein&doptcmdl=genpept&tool=mascot&term=150374325) from [Staphylococcus aureus subsp. aureus str. Newman](http://www.ncbi.nlm.nih.gov/Taxonomy/Browser/wwwtax.cgi?lvl=0&id=426430)

[gi|156721856](http://www.ncbi.nlm.nih.gov/entrez/query.fcgi?cmd=search&db=protein&doptcmdl=genpept&tool=mascot&term=156721856) from [Staphylococcus aureus subsp. aureus Mu3](http://www.ncbi.nlm.nih.gov/Taxonomy/Browser/wwwtax.cgi?lvl=0&id=418127)

[gi|160368377](http://www.ncbi.nlm.nih.gov/entrez/query.fcgi?cmd=search&db=protein&doptcmdl=genpept&tool=mascot&term=160368377) from [Staphylococcus aureus subsp. aureus USA300_TCH1516](http://www.ncbi.nlm.nih.gov/Taxonomy/Browser/wwwtax.cgi?lvl=0&id=451516)

[gi|253724230](http://www.ncbi.nlm.nih.gov/entrez/query.fcgi?cmd=search&db=protein&doptcmdl=genpept&tool=mascot&term=253724230) from [Staphylococcus aureus subsp. aureus USA300_TCH959](http://www.ncbi.nlm.nih.gov/Taxonomy/Browser/wwwtax.cgi?lvl=0&id=450394)

[gi|253728703](http://www.ncbi.nlm.nih.gov/entrez/query.fcgi?cmd=search&db=protein&doptcmdl=genpept&tool=mascot&term=253728703) from [Staphylococcus aureus subsp. aureus TCH130](http://www.ncbi.nlm.nih.gov/Taxonomy/Browser/wwwtax.cgi?lvl=0&id=548474)

[gi|257271923](http://www.ncbi.nlm.nih.gov/entrez/query.fcgi?cmd=search&db=protein&doptcmdl=genpept&tool=mascot&term=257271923) from [Staphylococcus aureus subsp. aureus 55/2053](http://www.ncbi.nlm.nih.gov/Taxonomy/Browser/wwwtax.cgi?lvl=0&id=585143)

[gi|257274967](http://www.ncbi.nlm.nih.gov/entrez/query.fcgi?cmd=search&db=protein&doptcmdl=genpept&tool=mascot&term=257274967) from [Staphylococcus aureus subsp. aureus 65-1322](http://www.ncbi.nlm.nih.gov/Taxonomy/Browser/wwwtax.cgi?lvl=0&id=585145)

[gi|257278885](http://www.ncbi.nlm.nih.gov/entrez/query.fcgi?cmd=search&db=protein&doptcmdl=genpept&tool=mascot&term=257278885) from [Staphylococcus aureus subsp. aureus 68-397](http://www.ncbi.nlm.nih.gov/Taxonomy/Browser/wwwtax.cgi?lvl=0&id=585146)

[gi|257281610](http://www.ncbi.nlm.nih.gov/entrez/query.fcgi?cmd=search&db=protein&doptcmdl=genpept&tool=mascot&term=257281610) from [Staphylococcus aureus subsp. aureus E1410](http://www.ncbi.nlm.nih.gov/Taxonomy/Browser/wwwtax.cgi?lvl=0&id=585153)

[gi|257284640](http://www.ncbi.nlm.nih.gov/entrez/query.fcgi?cmd=search&db=protein&doptcmdl=genpept&tool=mascot&term=257284640) from [Staphylococcus aureus subsp. aureus M876](http://www.ncbi.nlm.nih.gov/Taxonomy/Browser/wwwtax.cgi?lvl=0&id=585158)

[gi|257789515](http://www.ncbi.nlm.nih.gov/entrez/query.fcgi?cmd=search&db=protein&doptcmdl=genpept&tool=mascot&term=257789515) from [Staphylococcus aureus A9781](http://www.ncbi.nlm.nih.gov/Taxonomy/Browser/wwwtax.cgi?lvl=0&id=553596)

[gi|257839915](http://www.ncbi.nlm.nih.gov/entrez/query.fcgi?cmd=search&db=protein&doptcmdl=genpept&tool=mascot&term=257839915) from [Staphylococcus aureus A9763](http://www.ncbi.nlm.nih.gov/Taxonomy/Browser/wwwtax.cgi?lvl=0&id=553592)

[gi|257843490](http://www.ncbi.nlm.nih.gov/entrez/query.fcgi?cmd=search&db=protein&doptcmdl=genpept&tool=mascot&term=257843490) from [Staphylococcus aureus A9719](http://www.ncbi.nlm.nih.gov/Taxonomy/Browser/wwwtax.cgi?lvl=0&id=553588)

[gi|257846164](http://www.ncbi.nlm.nih.gov/entrez/query.fcgi?cmd=search&db=protein&doptcmdl=genpept&tool=mascot&term=257846164) from [Staphylococcus aureus A9635](http://www.ncbi.nlm.nih.gov/Taxonomy/Browser/wwwtax.cgi?lvl=0&id=553583)

[gi|257849047](http://www.ncbi.nlm.nih.gov/entrez/query.fcgi?cmd=search&db=protein&doptcmdl=genpept&tool=mascot&term=257849047) from [Staphylococcus aureus A9299](http://www.ncbi.nlm.nih.gov/Taxonomy/Browser/wwwtax.cgi?lvl=0&id=553581)

[gi|257850231](http://www.ncbi.nlm.nih.gov/entrez/query.fcgi?cmd=search&db=protein&doptcmdl=genpept&tool=mascot&term=257850231) from [Staphylococcus aureus A8115](http://www.ncbi.nlm.nih.gov/Taxonomy/Browser/wwwtax.cgi?lvl=0&id=553573)

[gi|257853618](http://www.ncbi.nlm.nih.gov/entrez/query.fcgi?cmd=search&db=protein&doptcmdl=genpept&tool=mascot&term=257853618) from [Staphylococcus aureus A6300](http://www.ncbi.nlm.nih.gov/Taxonomy/Browser/wwwtax.cgi?lvl=0&id=553571)

[gi|257857256](http://www.ncbi.nlm.nih.gov/entrez/query.fcgi?cmd=search&db=protein&doptcmdl=genpept&tool=mascot&term=257857256) from [Staphylococcus aureus A6224](http://www.ncbi.nlm.nih.gov/Taxonomy/Browser/wwwtax.cgi?lvl=0&id=553568)

[gi|257860128](http://www.ncbi.nlm.nih.gov/entrez/query.fcgi?cmd=search&db=protein&doptcmdl=genpept&tool=mascot&term=257860128) from [Staphylococcus aureus A5948](http://www.ncbi.nlm.nih.gov/Taxonomy/Browser/wwwtax.cgi?lvl=0&id=553567)

[gi|257863031](http://www.ncbi.nlm.nih.gov/entrez/query.fcgi?cmd=search&db=protein&doptcmdl=genpept&tool=mascot&term=257863031) from [Staphylococcus aureus A5937](http://www.ncbi.nlm.nih.gov/Taxonomy/Browser/wwwtax.cgi?lvl=0&id=553565)

[gi|259161556](http://www.ncbi.nlm.nih.gov/entrez/query.fcgi?cmd=search&db=protein&doptcmdl=genpept&tool=mascot&term=259161556) from [Staphylococcus aureus D30](http://www.ncbi.nlm.nih.gov/Taxonomy/Browser/wwwtax.cgi?lvl=0&id=455227)

[gi|262075315](http://www.ncbi.nlm.nih.gov/entrez/query.fcgi?cmd=search&db=protein&doptcmdl=genpept&tool=mascot&term=262075315) from [Staphylococcus aureus subsp. aureus ED98](http://www.ncbi.nlm.nih.gov/Taxonomy/Browser/wwwtax.cgi?lvl=0&id=681288)

[gi|269940897](http://www.ncbi.nlm.nih.gov/entrez/query.fcgi?cmd=search&db=protein&doptcmdl=genpept&tool=mascot&term=269940897) from [Staphylococcus aureus subsp. aureus TW20](http://www.ncbi.nlm.nih.gov/Taxonomy/Browser/wwwtax.cgi?lvl=0&id=663951)

[gi|282313682](http://www.ncbi.nlm.nih.gov/entrez/query.fcgi?cmd=search&db=protein&doptcmdl=genpept&tool=mascot&term=282313682) from [Staphylococcus aureus subsp. aureus C101](http://www.ncbi.nlm.nih.gov/Taxonomy/Browser/wwwtax.cgi?lvl=0&id=585149)

[gi|282316922](http://www.ncbi.nlm.nih.gov/entrez/query.fcgi?cmd=search&db=protein&doptcmdl=genpept&tool=mascot&term=282316922) from [Staphylococcus aureus subsp. aureus C427](http://www.ncbi.nlm.nih.gov/Taxonomy/Browser/wwwtax.cgi?lvl=0&id=585151)

[gi|282319155](http://www.ncbi.nlm.nih.gov/entrez/query.fcgi?cmd=search&db=protein&doptcmdl=genpept&tool=mascot&term=282319155) from [Staphylococcus aureus subsp. aureus D139](http://www.ncbi.nlm.nih.gov/Taxonomy/Browser/wwwtax.cgi?lvl=0&id=585152)

[gi|282322258](http://www.ncbi.nlm.nih.gov/entrez/query.fcgi?cmd=search&db=protein&doptcmdl=genpept&tool=mascot&term=282322258) from [Staphylococcus aureus subsp. aureus M899](http://www.ncbi.nlm.nih.gov/Taxonomy/Browser/wwwtax.cgi?lvl=0&id=585159)

[gi|282324677](http://www.ncbi.nlm.nih.gov/entrez/query.fcgi?cmd=search&db=protein&doptcmdl=genpept&tool=mascot&term=282324677) from [Staphylococcus aureus subsp. aureus WBG10049](http://www.ncbi.nlm.nih.gov/Taxonomy/Browser/wwwtax.cgi?lvl=0&id=585160)

[gi|282326982](http://www.ncbi.nlm.nih.gov/entrez/query.fcgi?cmd=search&db=protein&doptcmdl=genpept&tool=mascot&term=282326982) from [Staphylococcus aureus subsp. aureus WW2703/97](http://www.ncbi.nlm.nih.gov/Taxonomy/Browser/wwwtax.cgi?lvl=0&id=585161)

[gi|282331039](http://www.ncbi.nlm.nih.gov/entrez/query.fcgi?cmd=search&db=protein&doptcmdl=genpept&tool=mascot&term=282331039) from [Staphylococcus aureus subsp. aureus Btn1260](http://www.ncbi.nlm.nih.gov/Taxonomy/Browser/wwwtax.cgi?lvl=0&id=585148)

[gi|282590223](http://www.ncbi.nlm.nih.gov/entrez/query.fcgi?cmd=search&db=protein&doptcmdl=genpept&tool=mascot&term=282590223) from [Staphylococcus aureus A10102](http://www.ncbi.nlm.nih.gov/Taxonomy/Browser/wwwtax.cgi?lvl=0&id=553601)

[gi|282591856](http://www.ncbi.nlm.nih.gov/entrez/query.fcgi?cmd=search&db=protein&doptcmdl=genpept&tool=mascot&term=282591856) from [Staphylococcus aureus A9765](http://www.ncbi.nlm.nih.gov/Taxonomy/Browser/wwwtax.cgi?lvl=0&id=553594)

[gi|282595600](http://www.ncbi.nlm.nih.gov/entrez/query.fcgi?cmd=search&db=protein&doptcmdl=genpept&tool=mascot&term=282595600) from [Staphylococcus aureus subsp. aureus C160](http://www.ncbi.nlm.nih.gov/Taxonomy/Browser/wwwtax.cgi?lvl=0&id=585150)

[gi|282764892](http://www.ncbi.nlm.nih.gov/entrez/query.fcgi?cmd=search&db=protein&doptcmdl=genpept&tool=mascot&term=282764892) from [Staphylococcus aureus A8117](http://www.ncbi.nlm.nih.gov/Taxonomy/Browser/wwwtax.cgi?lvl=0&id=553574)

[gi|283460620](http://www.ncbi.nlm.nih.gov/entrez/query.fcgi?cmd=search&db=protein&doptcmdl=genpept&tool=mascot&term=283460620) from [Staphylococcus aureus subsp. aureus H19](http://www.ncbi.nlm.nih.gov/Taxonomy/Browser/wwwtax.cgi?lvl=0&id=585155)

[gi|283470617](http://www.ncbi.nlm.nih.gov/entrez/query.fcgi?cmd=search&db=protein&doptcmdl=genpept&tool=mascot&term=283470617) from [Staphylococcus aureus subsp. aureus ST398](http://www.ncbi.nlm.nih.gov/Taxonomy/Browser/wwwtax.cgi?lvl=0&id=523796)

[gi|283790313](http://www.ncbi.nlm.nih.gov/entrez/query.fcgi?cmd=search&db=protein&doptcmdl=genpept&tool=mascot&term=283790313) from [Staphylococcus aureus subsp. aureus A017934/97](http://www.ncbi.nlm.nih.gov/Taxonomy/Browser/wwwtax.cgi?lvl=0&id=585147)

[gi|285817080](http://www.ncbi.nlm.nih.gov/entrez/query.fcgi?cmd=search&db=protein&doptcmdl=genpept&tool=mascot&term=285817080) from [Staphylococcus aureus 04-02981](http://www.ncbi.nlm.nih.gov/Taxonomy/Browser/wwwtax.cgi?lvl=0&id=703339)

[gi|290920842](http://www.ncbi.nlm.nih.gov/entrez/query.fcgi?cmd=search&db=protein&doptcmdl=genpept&tool=mascot&term=290920842) from [Staphylococcus aureus subsp. aureus M1015](http://www.ncbi.nlm.nih.gov/Taxonomy/Browser/wwwtax.cgi?lvl=0&id=585156)

[gi|291096222](http://www.ncbi.nlm.nih.gov/entrez/query.fcgi?cmd=search&db=protein&doptcmdl=genpept&tool=mascot&term=291096222) from [Staphylococcus aureus subsp. aureus 58-424](http://www.ncbi.nlm.nih.gov/Taxonomy/Browser/wwwtax.cgi?lvl=0&id=585144)

[gi|291467122](http://www.ncbi.nlm.nih.gov/entrez/query.fcgi?cmd=search&db=protein&doptcmdl=genpept&tool=mascot&term=291467122) from [Staphylococcus aureus subsp. aureus M809](http://www.ncbi.nlm.nih.gov/Taxonomy/Browser/wwwtax.cgi?lvl=0&id=585157)

[gi|294822413](http://www.ncbi.nlm.nih.gov/entrez/query.fcgi?cmd=search&db=protein&doptcmdl=genpept&tool=mascot&term=294822413) from [Staphylococcus aureus A9754](http://www.ncbi.nlm.nih.gov/Taxonomy/Browser/wwwtax.cgi?lvl=0&id=553590)

[gi|294968937](http://www.ncbi.nlm.nih.gov/entrez/query.fcgi?cmd=search&db=protein&doptcmdl=genpept&tool=mascot&term=294968937) from [Staphylococcus aureus A8819](http://www.ncbi.nlm.nih.gov/Taxonomy/Browser/wwwtax.cgi?lvl=0&id=553580)

[gi|295128273](http://www.ncbi.nlm.nih.gov/entrez/query.fcgi?cmd=search&db=protein&doptcmdl=genpept&tool=mascot&term=295128273) from [Staphylococcus aureus subsp. aureus EMRSA16](http://www.ncbi.nlm.nih.gov/Taxonomy/Browser/wwwtax.cgi?lvl=0&id=585154)

[gi|296887411](http://www.ncbi.nlm.nih.gov/entrez/query.fcgi?cmd=search&db=protein&doptcmdl=genpept&tool=mascot&term=296887411) from [Staphylococcus aureus subsp. aureus ATCC 51811](http://www.ncbi.nlm.nih.gov/Taxonomy/Browser/wwwtax.cgi?lvl=0&id=762962)

[gi|297178608](http://www.ncbi.nlm.nih.gov/entrez/query.fcgi?cmd=search&db=protein&doptcmdl=genpept&tool=mascot&term=297178608) from [Staphylococcus aureus A8796](http://www.ncbi.nlm.nih.gov/Taxonomy/Browser/wwwtax.cgi?lvl=0&id=553577)

[gi|297576010](http://www.ncbi.nlm.nih.gov/entrez/query.fcgi?cmd=search&db=protein&doptcmdl=genpept&tool=mascot&term=297576010) from [Staphylococcus aureus subsp. aureus MN8](http://www.ncbi.nlm.nih.gov/Taxonomy/Browser/wwwtax.cgi?lvl=0&id=548470)

[gi|298694697](http://www.ncbi.nlm.nih.gov/entrez/query.fcgi?cmd=search&db=protein&doptcmdl=genpept&tool=mascot&term=298694697) from [Staphylococcus aureus subsp. aureus ED133](http://www.ncbi.nlm.nih.gov/Taxonomy/Browser/wwwtax.cgi?lvl=0&id=685039)

[gi|300886273](http://www.ncbi.nlm.nih.gov/entrez/query.fcgi?cmd=search&db=protein&doptcmdl=genpept&tool=mascot&term=300886273) from [Staphylococcus aureus subsp. aureus TCH70](http://www.ncbi.nlm.nih.gov/Taxonomy/Browser/wwwtax.cgi?lvl=0&id=548475)

[gi|302333015](http://www.ncbi.nlm.nih.gov/entrez/query.fcgi?cmd=search&db=protein&doptcmdl=genpept&tool=mascot&term=302333015) from [Staphylococcus aureus subsp. aureus JKD6159](http://www.ncbi.nlm.nih.gov/Taxonomy/Browser/wwwtax.cgi?lvl=0&id=869816)

[gi|302751231](http://www.ncbi.nlm.nih.gov/entrez/query.fcgi?cmd=search&db=protein&doptcmdl=genpept&tool=mascot&term=302751231) from [Staphylococcus aureus subsp. aureus str. JKD6008](http://www.ncbi.nlm.nih.gov/Taxonomy/Browser/wwwtax.cgi?lvl=0&id=546342)

[gi|304340437](http://www.ncbi.nlm.nih.gov/entrez/query.fcgi?cmd=search&db=protein&doptcmdl=genpept&tool=mascot&term=304340437) from [Staphylococcus aureus subsp. aureus ATCC BAA-39](http://www.ncbi.nlm.nih.gov/Taxonomy/Browser/wwwtax.cgi?lvl=0&id=862516)

[gi|312438198](http://www.ncbi.nlm.nih.gov/entrez/query.fcgi?cmd=search&db=protein&doptcmdl=genpept&tool=mascot&term=312438198) from [Staphylococcus aureus subsp. aureus TCH60](http://www.ncbi.nlm.nih.gov/Taxonomy/Browser/wwwtax.cgi?lvl=0&id=548473)

[gi|312829797](http://www.ncbi.nlm.nih.gov/entrez/query.fcgi?cmd=search&db=protein&doptcmdl=genpept&tool=mascot&term=312829797) from [Staphylococcus aureus subsp. aureus ECT-R 2](http://www.ncbi.nlm.nih.gov/Taxonomy/Browser/wwwtax.cgi?lvl=0&id=889933)

[gi|315131210](http://www.ncbi.nlm.nih.gov/entrez/query.fcgi?cmd=search&db=protein&doptcmdl=genpept&tool=mascot&term=315131210) from [Staphylococcus aureus subsp. aureus CGS03](http://www.ncbi.nlm.nih.gov/Taxonomy/Browser/wwwtax.cgi?lvl=0&id=543540)

[gi|315195294](http://www.ncbi.nlm.nih.gov/entrez/query.fcgi?cmd=search&db=protein&doptcmdl=genpept&tool=mascot&term=315195294) from [Staphylococcus aureus subsp. aureus CGS00](http://www.ncbi.nlm.nih.gov/Taxonomy/Browser/wwwtax.cgi?lvl=0&id=543538)

[gi|315195914](http://www.ncbi.nlm.nih.gov/entrez/query.fcgi?cmd=search&db=protein&doptcmdl=genpept&tool=mascot&term=315195914) from [Staphylococcus aureus subsp. aureus CGS01](http://www.ncbi.nlm.nih.gov/Taxonomy/Browser/wwwtax.cgi?lvl=0&id=543539)

[gi|320140790](http://www.ncbi.nlm.nih.gov/entrez/query.fcgi?cmd=search&db=protein&doptcmdl=genpept&tool=mascot&term=320140790) from [Staphylococcus aureus subsp. aureus MRSA131](http://www.ncbi.nlm.nih.gov/Taxonomy/Browser/wwwtax.cgi?lvl=0&id=754025)

[gi|320142182](http://www.ncbi.nlm.nih.gov/entrez/query.fcgi?cmd=search&db=protein&doptcmdl=genpept&tool=mascot&term=320142182) from [Staphylococcus aureus subsp. aureus MRSA177](http://www.ncbi.nlm.nih.gov/Taxonomy/Browser/wwwtax.cgi?lvl=0&id=754026)

[gi|323438687](http://www.ncbi.nlm.nih.gov/entrez/query.fcgi?cmd=search&db=protein&doptcmdl=genpept&tool=mascot&term=323438687) (no taxonomy information for this entry)

[gi|323444056](http://www.ncbi.nlm.nih.gov/entrez/query.fcgi?cmd=search&db=protein&doptcmdl=genpept&tool=mascot&term=323444056) (no taxonomy information for this entry)

Fixed modifications: Carbamidomethyl (C)

Variable modifications: Oxidation (M)

Cleavage by Trypsin: cuts C-term side of KR unless next residue is P

Sequence Coverage: **39%**

Matched peptides shown in **Bold Red**

**1** MKQGTVKWFN AEKGFGFIEV EGENDVFVHF SAINQDGYK**S LEEGQAVEFE**

**51 VVEGDRGPQA ANVVK**L

  Residue Number  Increasing Mass  Decreasing Mass

**Start - End Observed Mr(expt) Mr(calc) ppm Miss Sequence**

**40 - 65 2757.8882 2756.8809 2756.3617 188 1 K.SLEEGQAVEFEVVEGDRGPQAANVVK.L**  ([Ions score 104](http://www.matrixscience.com/cgi/peptide_view.pl?file=../data/20110224/FttoIeSte.dat&query=7&hit=1&index=gi|15924392&px=1&section=5&ave_thresh=50&_ignoreionsscorebelow=0&report=20&_sigthreshold=0.05&_msresflags=1025&_msresflags2=2&percolate=-1&percolate_rt=0))

### Search Parameters

**Type of search : MS/MS Ion Search**

**Enzyme : Trypsin**

**Fixed modifications :** [**Carbamidomethyl (C)**](http://www.matrixscience.com/cgi/client.pl?modification&mod_name=Carbamidomethyl+(C)&file=../data/20110224/FttoIeSte.dat)

**Variable modifications :** [**Oxidation (M)**](http://www.matrixscience.com/cgi/client.pl?modification&mod_name=Oxidation+(M)&file=../data/20110224/FttoIeSte.dat)

**Mass values : Monoisotopic**

**Protein Mass : Unrestricted**

**Peptide Mass Tolerance : ± 189 ppm**

**Fragment Mass Tolerance: ± 2 Da**

**Max Missed Cleavages : 1**

**Instrument type : MALDI-TOF-TOF**

**Number of queries : 8**
