# Supplementary material for: Isolation of Two Strong Poly (U) Binding Proteins from Moderate Halophile Halomonas eurihalina and Their Identification as Cold Shock Proteins
Source: PLoS One. 2012 Apr 13;7(4):e34409. doi: 10.1371/journal.pone.0034409 (PMC3326018; doi:10.1371/journal.pone.0034409)
Supplement: File S2 — Peptide Mass Fingerprinting and MASCOT Search results of 8 kDa protein. (DOC) [file pone.0034409.s002.doc]

File S2

Peptide Mass Fingerprinting and MASCOT Search results of 8 kDa protein (spot No. 2)

**
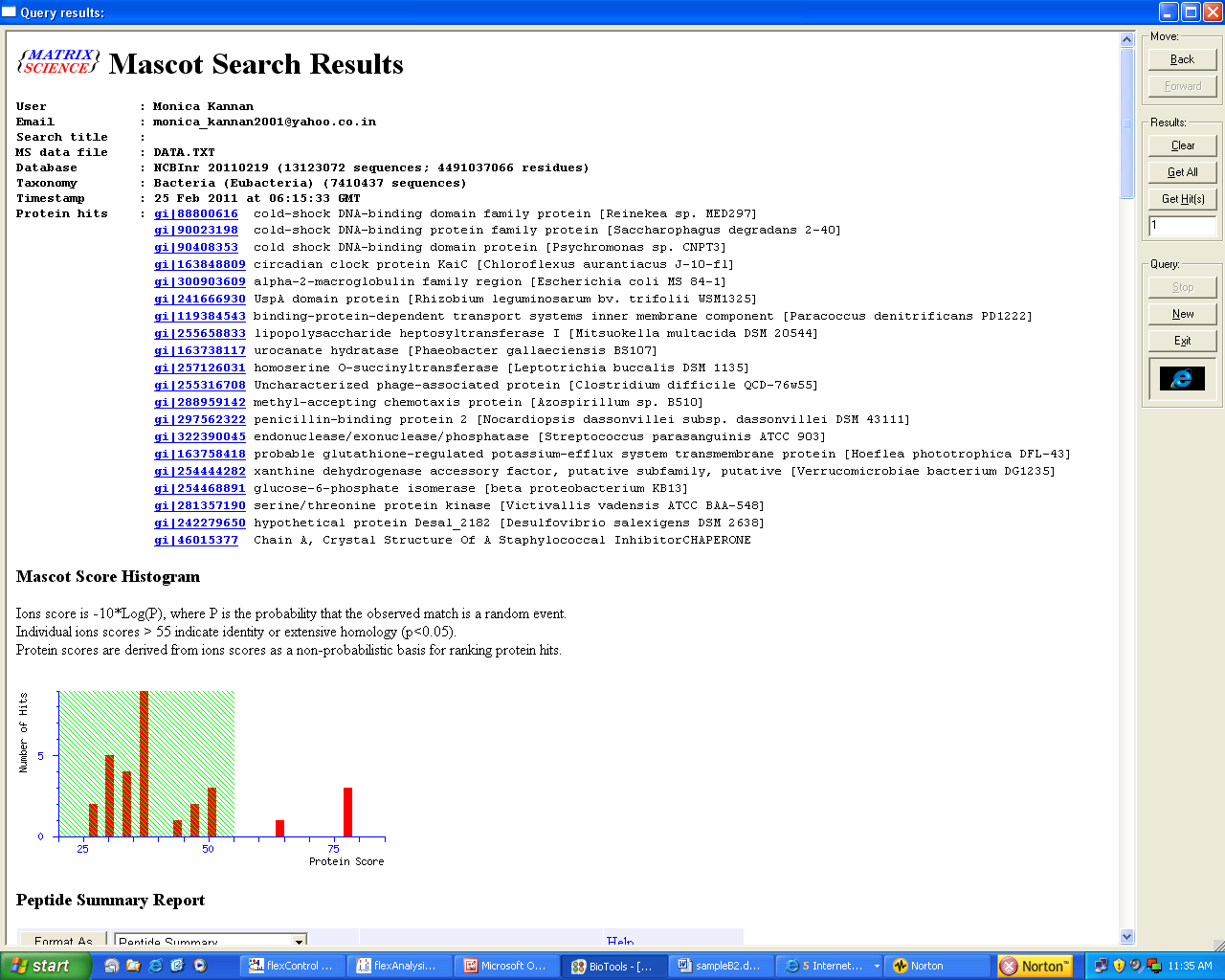
**

| **1.** | [gi|88800616](http://www.matrixscience.com/cgi/protein_view.pl?file=../data/20110225/FttoCfTEt.dat&hit=gi|88800616&db_idx=1&px=1&ave_thresh=55&_ignoreionsscorebelow=0&report=20&_sigthreshold=0.05&_msresflags=1025&_msresflags2=2&percolate=-1&percolate_rt=0)    **Mass:** 7275     **Score:** 78     **Matches:** 1(1)  **Sequences:** 1(1) |
| --- | --- |
|  | cold-shock DNA-binding domain family protein [Reinekea sp. MED297] |

|  | Check to include this hit in error tolerant search |
| --- | --- |
|  |  |

|  | **Query** | | **Observed** | **Mr(expt)** | **Mr(calc)** | **ppm** | **Miss** | **Score** | **Expect** | **Rank** | | **Unique** | **Peptide** |
| --- | --- | --- | --- | --- | --- | --- | --- | --- | --- | --- | --- | --- | --- |
|  | [2](http://www.matrixscience.com/cgi/peptide_view.pl?file=../data/20110225/FttoCfTEt.dat&query=2&hit=1&index=gi|88800616&px=1&section=5&ave_thresh=55&_ignoreionsscorebelow=0&report=20&_sigthreshold=0.05&_msresflags=1025&_msresflags2=2&percolate=-1&percolate_rt=0) | | **1863.8481** | **1862.8408** | **1862.9738** | **-71.38** | **1** | **78** | **0.00031** | **1** | | **U** | **K.TLAEGQKVEFTVTQGQK.G** |
|  | | | | | | | | | | |  | | |
|  | | **Proteins matching the same set of peptides:** | | | | | | | | |  | | |

|  | [gi|90408760](http://www.matrixscience.com/cgi/protein_view.pl?file=../data/20110225/FttoCfTEt.dat&hit=gi|90408760&db_idx=1&px=1&ave_thresh=55&_ignoreionsscorebelow=0&report=20&_sigthreshold=0.05&_msresflags=1025&_msresflags2=2&percolate=-1&percolate_rt=0)    **Mass:** 7359     **Score:** 78     **Matches:** 1(1)  **Sequences:** 1(1) |
| --- | --- |
|  | Cold shock protein [Psychromonas sp. CNPT3] |

|  | [gi|109897864](http://www.matrixscience.com/cgi/protein_view.pl?file=../data/20110225/FttoCfTEt.dat&hit=gi|109897864&db_idx=1&px=1&ave_thresh=55&_ignoreionsscorebelow=0&report=20&_sigthreshold=0.05&_msresflags=1025&_msresflags2=2&percolate=-1&percolate_rt=0)    **Mass:** 7404     **Score:** 78     **Matches:** 1(1)  **Sequences:** 1(1) |
| --- | --- |
|  | cold-shock DNA-binding domain-containing protein [Pseudoalteromonas atlantica T6c] |

|  | [gi|119475889](http://www.matrixscience.com/cgi/protein_view.pl?file=../data/20110225/FttoCfTEt.dat&hit=gi|119475889&db_idx=1&px=1&ave_thresh=55&_ignoreionsscorebelow=0&report=20&_sigthreshold=0.05&_msresflags=1025&_msresflags2=2&percolate=-1&percolate_rt=0)    **Mass:** 7492     **Score:** 78     **Matches:** 1(1)  **Sequences:** 1(1) |
| --- | --- |
|  | Cold shock protein [marine gamma proteobacterium HTCC2143] |

|  | [gi|119945576](http://www.matrixscience.com/cgi/protein_view.pl?file=../data/20110225/FttoCfTEt.dat&hit=gi|119945576&db_idx=1&px=1&ave_thresh=55&_ignoreionsscorebelow=0&report=20&_sigthreshold=0.05&_msresflags=1025&_msresflags2=2&percolate=-1&percolate_rt=0)    **Mass:** 7390     **Score:** 78     **Matches:** 1(1)  **Sequences:** 1(1) |
| --- | --- |
|  | cold-shock DNA-binding domain-containing protein [Psychromonas ingrahamii 37] |

| **2.** | [gi|90023198](http://www.matrixscience.com/cgi/protein_view.pl?file=../data/20110225/FttoCfTEt.dat&hit=gi|90023198&db_idx=1&px=1&ave_thresh=55&_ignoreionsscorebelow=0&report=20&_sigthreshold=0.05&_msresflags=1025&_msresflags2=2&percolate=-1&percolate_rt=0)    **Mass:** 7433     **Score:** 78     **Matches:** 1(1)  **Sequences:** 1(1) |
| --- | --- |
|  | cold-shock DNA-binding protein family protein [Saccharophagus degradans 2-40] |

|  | Check to include this hit in error tolerant search |
| --- | --- |
|  |  |

|  | **Query** | **Observed** | **Mr(expt)** | **Mr(calc)** | **ppm** | **Miss** | **Score** | **Expect** | **Rank** | **Unique** | **Peptide** |
| --- | --- | --- | --- | --- | --- | --- | --- | --- | --- | --- | --- |
|  | [2](http://www.matrixscience.com/cgi/peptide_view.pl?file=../data/20110225/FttoCfTEt.dat&query=2&hit=3&index=gi|90023198&px=1&section=5&ave_thresh=55&_ignoreionsscorebelow=0&report=20&_sigthreshold=0.05&_msresflags=1025&_msresflags2=2&percolate=-1&percolate_rt=0) | 1863.8481 | 1862.8408 | 1862.9374 | -51.85 | 0 | 78 | 0.00031 | 1 | U | K.TLAEGQQVEFTVTQGQK.G |

|  | |
| --- | --- |
|  | **Proteins matching the same set of peptides:** |

|  | [gi|90023400](http://www.matrixscience.com/cgi/protein_view.pl?file=../data/20110225/FttoCfTEt.dat&hit=gi|90023400&db_idx=1&px=1&ave_thresh=55&_ignoreionsscorebelow=0&report=20&_sigthreshold=0.05&_msresflags=1025&_msresflags2=2&percolate=-1&percolate_rt=0)    **Mass:** 7380     **Score:** 78     **Matches:** 1(1)  **Sequences:** 1(1) |
| --- | --- |
|  | cold-shock DNA-binding protein family protein [Saccharophagus degradans 2-40] |

|  | [gi|120554260](http://www.matrixscience.com/cgi/protein_view.pl?file=../data/20110225/FttoCfTEt.dat&hit=gi|120554260&db_idx=1&px=1&ave_thresh=55&_ignoreionsscorebelow=0&report=20&_sigthreshold=0.05&_msresflags=1025&_msresflags2=2&percolate=-1&percolate_rt=0)    **Mass:** 7209     **Score:** 78     **Matches:** 1(1)  **Sequences:** 1(1) |
| --- | --- |
|  | cold-shock DNA-binding domain-containing protein [Marinobacter aquaeolei VT8] |

|  | [gi|120556517](http://www.matrixscience.com/cgi/protein_view.pl?file=../data/20110225/FttoCfTEt.dat&hit=gi|120556517&db_idx=1&px=1&ave_thresh=55&_ignoreionsscorebelow=0&report=20&_sigthreshold=0.05&_msresflags=1025&_msresflags2=2&percolate=-1&percolate_rt=0)    **Mass:** 7239     **Score:** 78     **Matches:** 1(1)  **Sequences:** 1(1) |
| --- | --- |
|  | cold-shock DNA-binding domain-containing protein [Marinobacter aquaeolei VT8] |

|  | [gi|126664472](http://www.matrixscience.com/cgi/protein_view.pl?file=../data/20110225/FttoCfTEt.dat&hit=gi|126664472&db_idx=1&px=1&ave_thresh=55&_ignoreionsscorebelow=0&report=20&_sigthreshold=0.05&_msresflags=1025&_msresflags2=2&percolate=-1&percolate_rt=0)    **Mass:** 7209     **Score:** 78     **Matches:** 1(1)  **Sequences:** 1(1) |
| --- | --- |
|  | cold-shock DNA-binding domain family protein [Marinobacter sp. ELB17] |

|  | [gi|126664621](http://www.matrixscience.com/cgi/protein_view.pl?file=../data/20110225/FttoCfTEt.dat&hit=gi|126664621&db_idx=1&px=1&ave_thresh=55&_ignoreionsscorebelow=0&report=20&_sigthreshold=0.05&_msresflags=1025&_msresflags2=2&percolate=-1&percolate_rt=0)    **Mass:** 7235     **Score:** 78     **Matches:** 1(1)  **Sequences:** 1(1) |
| --- | --- |
|  | cold-shock DNA-binding domain family protein [Marinobacter sp. ELB17] |

|  | [gi|149377667](http://www.matrixscience.com/cgi/protein_view.pl?file=../data/20110225/FttoCfTEt.dat&hit=gi|149377667&db_idx=1&px=1&ave_thresh=55&_ignoreionsscorebelow=0&report=20&_sigthreshold=0.05&_msresflags=1025&_msresflags2=2&percolate=-1&percolate_rt=0)    **Mass:** 7241     **Score:** 78     **Matches:** 1(1)  **Sequences:** 1(1) |
| --- | --- |
|  | cold-shock DNA-binding domain family protein [Marinobacter algicola DG893] |

|  | [gi|311693752](http://www.matrixscience.com/cgi/protein_view.pl?file=../data/20110225/FttoCfTEt.dat&hit=gi|311693752&db_idx=1&px=1&ave_thresh=55&_ignoreionsscorebelow=0&report=20&_sigthreshold=0.05&_msresflags=1025&_msresflags2=2&percolate=-1&percolate_rt=0)    **Mass:** 8235     **Score:** 78     **Matches:** 1(1)  **Sequences:** 1(1) |
| --- | --- |
|  | cold shock, CspA [marine bacterium HP15] |

| **3.** | [gi|90408353](http://www.matrixscience.com/cgi/protein_view.pl?file=../data/20110225/FttoCfTEt.dat&hit=gi|90408353&db_idx=1&px=1&ave_thresh=55&_ignoreionsscorebelow=0&report=20&_sigthreshold=0.05&_msresflags=1025&_msresflags2=2&percolate=-1&percolate_rt=0)    **Mass:** 7449     **Score:** 78     **Matches:** 1(1)  **Sequences:** 1(1) |
| --- | --- |
|  | cold shock DNA-binding domain protein [Psychromonas sp. CNPT3] |

|  | Check to include this hit in error tolerant search |
| --- | --- |
|  |  |

|  | **Query** | **Observed** | **Mr(expt)** | **Mr(calc)** | **ppm** | **Miss** | **Score** | **Expect** | **Rank** | **Unique** | **Peptide** |
| --- | --- | --- | --- | --- | --- | --- | --- | --- | --- | --- | --- |
|  | [2](http://www.matrixscience.com/cgi/peptide_view.pl?file=../data/20110225/FttoCfTEt.dat&query=2&hit=2&index=gi|90408353&px=1&section=5&ave_thresh=55&_ignoreionsscorebelow=0&report=20&_sigthreshold=0.05&_msresflags=1025&_msresflags2=2&percolate=-1&percolate_rt=0) | 1863.8481 | 1862.8408 | 1861.9898 | 457 | 1 | 78 | 0.00031 | 1 | U | K.TLAEGQKVQFTVTQGQK.G |

|  | |
| --- | --- |
|  | **Proteins matching the same set of peptides:** |

|  | [gi|145297910](http://www.matrixscience.com/cgi/protein_view.pl?file=../data/20110225/FttoCfTEt.dat&hit=gi|145297910&db_idx=1&px=1&ave_thresh=55&_ignoreionsscorebelow=0&report=20&_sigthreshold=0.05&_msresflags=1025&_msresflags2=2&percolate=-1&percolate_rt=0)    **Mass:** 7633     **Score:** 78     **Matches:** 1(1)  **Sequences:** 1(1) |
| --- | --- |
|  | cold shock protein [Aeromonas salmonicida subsp. salmonicida A449] |

### Protein View

Top of Form

Match to: **gi|88800616** Score: **78**

**cold-shock DNA-binding domain family protein [Reinekea sp. MED297]**

Found in search of DATA.TXT

Nominal mass (M_r_): **7275**; Calculated pI value: **5.63**

NCBI BLAST search of [gi|88800616](http://www.ncbi.nlm.nih.gov/blast/Blast.cgi?ALIGNMENTS=50&ALIGNMENT_VIEW=Pairwise&AUTO_FORMAT=Semiauto&CDD_SEARCH=on&CLIENT=web&COMPOSITION_BASED_STATISTICS=on&DATABASE=nr&DESCRIPTIONS=100&ENTREZ_QUERY=(none)&EXPECT=10&FILTER=L&FORMAT_BLOCK_ON_RESPAGE=None&FORMAT_OBJECT=Alignment&FORMAT_TYPE=HTML&GAPCOSTS=11+1&I_THRESH=0.001&LAYOUT=TwoWindows&MATRIX_NAME=BLOSUM62&NCBI_GI=on&PAGE=Proteins&PROGRAM=blastp&QUERY=MSTVTGTVKWFNEAKGFGFIEQQNGPDVFAHFSAITGSGFKTLAEGQKVEFTVTQGQKGPQAENIVAI&SERVICE=plain&SET_DEFAULTS.x=9&SET_DEFAULTS.y=5&SHOW_OVERVIEW=on&WORD_SIZE=3&END_OF_HTTPGET=Yes) against nr

Unformatted [sequence string](http://www.matrixscience.com/cgi/getseq.pl?NCBInr+gi|88800616+seq) for pasting into other applications

Taxonomy: [Reinekea blandensis MED297](http://www.ncbi.nlm.nih.gov/Taxonomy/Browser/wwwtax.cgi?lvl=0&id=314283)

Links to retrieve other entries containing this sequence from NCBI Entrez:

[gi|88776671](http://www.ncbi.nlm.nih.gov/entrez/query.fcgi?cmd=search&db=protein&doptcmdl=genpept&tool=mascot&term=88776671) from [Reinekea blandensis MED297](http://www.ncbi.nlm.nih.gov/Taxonomy/Browser/wwwtax.cgi?lvl=0&id=314283)

Fixed modifications: Carbamidomethyl (C)

Variable modifications: Oxidation (M)

Cleavage by Trypsin: cuts C-term side of KR unless next residue is P

Sequence Coverage: **25%**

Matched peptides shown in **Bold Red**

**1** MSTVTGTVKW FNEAKGFGFI EQQNGPDVFA HFSAITGSGF K**TLAEGQKVE**

**51 FTVTQGQK**GP QAENIVAI

  Residue Number  Increasing Mass  Decreasing Mass

**Start - End Observed Mr(expt) Mr(calc) ppm Miss Sequence**

**42 - 58 1863.8481 1862.8408 1862.9738 -71 1 K.TLAEGQKVEFTVTQGQK.G**  ([Ions score 78](http://www.matrixscience.com/cgi/peptide_view.pl?file=../data/20110225/FttoCfTEt.dat&query=2&hit=1&index=gi|88800616&px=1&section=5&ave_thresh=55&_ignoreionsscorebelow=0&report=20&_sigthreshold=0.05&_msresflags=1025&_msresflags2=2&percolate=-1&percolate_rt=0))

### Search Parameters

**Type of search : MS/MS Ion Search**

**Enzyme : Trypsin**

**Fixed modifications :** [**Carbamidomethyl (C)**](http://www.matrixscience.com/cgi/client.pl?modification&mod_name=Carbamidomethyl+(C)&file=../data/20110225/FttoCfTEt.dat)

**Variable modifications :** [**Oxidation (M)**](http://www.matrixscience.com/cgi/client.pl?modification&mod_name=Oxidation+(M)&file=../data/20110225/FttoCfTEt.dat)

**Mass values : Monoisotopic**

**Protein Mass : Unrestricted**

**Peptide Mass Tolerance : ± 580 ppm**

**Fragment Mass Tolerance: ± 2 Da**

**Max Missed Cleavages : 1**

**Instrument type : MALDI-TOF-TOF**

**Number of queries : 3**
