# Supplementary material for: Isolation of Two Strong Poly (U) Binding Proteins from Moderate Halophile Halomonas eurihalina and Their Identification as Cold Shock Proteins
Source: PLoS One. 2012 Apr 13;7(4):e34409. doi: 10.1371/journal.pone.0034409 (PMC3326018; doi:10.1371/journal.pone.0034409)
Supplement: File S3 — Bioinformatic analysis for finding homologues of 12 kDa and 8 kDa cold shock proteins of Halomonas eurihalina. (DOC) [file pone.0034409.s003.doc]

## File S3

## Bioinformatic analysis for finding homologues of 12 kDa and 8 kDa cold shock proteins of *Halomonas eurihalina*

## Blast searches were performed using 28 amino acid residue peptide for 12 kDa protein and 19 amino acid peptide for 8 kDa protein (Table 1 of Manuscript)

## 1. Blast search of KEGG genome database

## BLASTP Search Result *Halomonas eurihalina* 12 kDa cold shock protein

**Database: genes:**

**Protein sequence database entries related to query** - 500 hits

Top of Form

command: blastp

query: /share/www/blast/120120152928c0IKM/query.seq

database: genes (prot)

------------------------

command query database

blastp prot prot

Entry bits E-val

--------------------------------------------------------------------- -----------

[sab:SAB1258c](http://www.genome.jp/dbget-bin/www_bget?sab:SAB1258c) cspA; cold shock protein; K03704 cold shock protein... [57](http://www.genome.jp/tmp/blast/120120152928c0IKM/result_blast.html#sab:SAB1258c) 6e-07

[sad:SAAV_1386](http://www.genome.jp/dbget-bin/www_bget?sad:SAAV_1386) CSD family cold shock protein; K03704 cold shock p... [57](http://www.genome.jp/tmp/blast/120120152928c0IKM/result_blast.html#sad:SAAV_1386) 6e-07

[sae:NWMN_1313](http://www.genome.jp/dbget-bin/www_bget?sae:NWMN_1313) cspA; major cold-shock protein CspA; K03704 cold s... [57](http://www.genome.jp/tmp/blast/120120152928c0IKM/result_blast.html#sae:NWMN_1313) 6e-07

[sao:SAOUHSC_01403](http://www.genome.jp/dbget-bin/www_bget?sao:SAOUHSC_01403) cold shock protein; K03704 cold shock protein ... [57](http://www.genome.jp/tmp/blast/120120152928c0IKM/result_blast.html#sao:SAOUHSC_01403) 6e-07

[saa:SAUSA300_1295](http://www.genome.jp/dbget-bin/www_bget?saa:SAUSA300_1295) CSD family cold shock protein; K03704 cold sho... [57](http://www.genome.jp/tmp/blast/120120152928c0IKM/result_blast.html#saa:SAUSA300_1295) 6e-07

[sax:USA300HOU_1337](http://www.genome.jp/dbget-bin/www_bget?sax:USA300HOU_1337) cspA; cold shock protein CspA; K03704 cold sh... [57](http://www.genome.jp/tmp/blast/120120152928c0IKM/result_blast.html#sax:USA300HOU_1337) 6e-07

[sac:SACOL1437](http://www.genome.jp/dbget-bin/www_bget?sac:SACOL1437) CSD family cold shock protein; K03704 cold shock p... [57](http://www.genome.jp/tmp/blast/120120152928c0IKM/result_blast.html#sac:SACOL1437) 6e-07

[sar:SAR1414](http://www.genome.jp/dbget-bin/www_bget?sar:SAR1414) cspA; cold shock protein; K03704 cold shock protein ... [57](http://www.genome.jp/tmp/blast/120120152928c0IKM/result_blast.html#sar:SAR1414) 6e-07

[sas:SAS1343](http://www.genome.jp/dbget-bin/www_bget?sas:SAS1343) cold shock protein; K03704 cold shock protein (beta-... [57](http://www.genome.jp/tmp/blast/120120152928c0IKM/result_blast.html#sas:SAS1343) 6e-07

[sam:MW1290](http://www.genome.jp/dbget-bin/www_bget?sam:MW1290) cspA; major cold shock protein CspA; K03704 cold shoc... [57](http://www.genome.jp/tmp/blast/120120152928c0IKM/result_blast.html#sam:MW1290) 6e-07

[saj:SaurJH9_1463](http://www.genome.jp/dbget-bin/www_bget?saj:SaurJH9_1463) cold-shock DNA-binding domain-containing protei... [57](http://www.genome.jp/tmp/blast/120120152928c0IKM/result_blast.html#saj:SaurJH9_1463) 6e-07

[sah:SaurJH1_1492](http://www.genome.jp/dbget-bin/www_bget?sah:SaurJH1_1492) DNA-binding cold-shock protein; K03704 cold sho... [57](http://www.genome.jp/tmp/blast/120120152928c0IKM/result_blast.html#sah:SaurJH1_1492) 6e-07

[saw:SAHV_1390](http://www.genome.jp/dbget-bin/www_bget?saw:SAHV_1390) cspA; major cold shock protein; K03704 cold shock ... [57](http://www.genome.jp/tmp/blast/120120152928c0IKM/result_blast.html#saw:SAHV_1390) 6e-07

[sav:SAV1402](http://www.genome.jp/dbget-bin/www_bget?sav:SAV1402) cspA; major cold shock protein; K03704 cold shock pr... [57](http://www.genome.jp/tmp/blast/120120152928c0IKM/result_blast.html#sav:SAV1402) 6e-07

[sau:SA1234](http://www.genome.jp/dbget-bin/www_bget?sau:SA1234) cspA; major cold shock protein CspA; K03704 cold shoc... [57](http://www.genome.jp/tmp/blast/120120152928c0IKM/result_blast.html#sau:SA1234) 6e-07

[sca:Sca_1045](http://www.genome.jp/dbget-bin/www_bget?sca:Sca_1045) csp; putative cold shock protein; K03704 cold shock... [57](http://www.genome.jp/tmp/blast/120120152928c0IKM/result_blast.html#sca:Sca_1045) 6e-07

[ssp:SSP2312](http://www.genome.jp/dbget-bin/www_bget?ssp:SSP2312) cold shock protein; K03704 cold shock protein (beta-... [56](http://www.genome.jp/tmp/blast/120120152928c0IKM/result_blast.html#ssp:SSP2312) 7e-07

[ser:SERP0972](http://www.genome.jp/dbget-bin/www_bget?ser:SERP0972) CSD family cold shock protein; K03704 cold shock pr... [55](http://www.genome.jp/tmp/blast/120120152928c0IKM/result_blast.html#ser:SERP0972) 1e-06

[slg:SLGD_01496](http://www.genome.jp/dbget-bin/www_bget?slg:SLGD_01496) cold shock protein CspA; K03704 cold shock protei... [55](http://www.genome.jp/tmp/blast/120120152928c0IKM/result_blast.html#slg:SLGD_01496) 1e-06

[sha:SH1509](http://www.genome.jp/dbget-bin/www_bget?sha:SH1509) cspA; major cold shock protein CspA; K03704 cold shoc... [55](http://www.genome.jp/tmp/blast/120120152928c0IKM/result_blast.html#sha:SH1509) 1e-06

[ssp:SSP1349](http://www.genome.jp/dbget-bin/www_bget?ssp:SSP1349) cold shock protein; K03704 cold shock protein (beta-... [55](http://www.genome.jp/tmp/blast/120120152928c0IKM/result_blast.html#ssp:SSP1349) 1e-06

[sep:SE1086](http://www.genome.jp/dbget-bin/www_bget?sep:SE1086) major cold shock protein CspA; K03704 cold shock prot... [55](http://www.genome.jp/tmp/blast/120120152928c0IKM/result_blast.html#sep:SE1086) 1e-06

[ssd:SPSINT_1112](http://www.genome.jp/dbget-bin/www_bget?ssd:SPSINT_1112) Cold shock protein CspA; K03704 cold shock prote... [54](http://www.genome.jp/tmp/blast/120120152928c0IKM/result_blast.html#ssd:SPSINT_1112) 3e-06

[bpf:BpOF4_15925](http://www.genome.jp/dbget-bin/www_bget?bpf:BpOF4_15925) cspA; cold shock protein; K03704 cold shock prot... [54](http://www.genome.jp/tmp/blast/120120152928c0IKM/result_blast.html#bpf:BpOF4_15925) 5e-06

[has:Halsa_2151](http://www.genome.jp/dbget-bin/www_bget?has:Halsa_2151) cold-shock protein; K03704 cold shock protein (be... [52](http://www.genome.jp/tmp/blast/120120152928c0IKM/result_blast.html#has:Halsa_2151) 1e-05

[ppy:PPE_01697](http://www.genome.jp/dbget-bin/www_bget?ppy:PPE_01697) Cold shock-like protein cspD; K03704 cold shock pr... [52](http://www.genome.jp/tmp/blast/120120152928c0IKM/result_blast.html#ppy:PPE_01697) 1e-05

[bco:Bcell_0298](http://www.genome.jp/dbget-bin/www_bget?bco:Bcell_0298) cold-shock DNA-binding domain-containing protein;... [52](http://www.genome.jp/tmp/blast/120120152928c0IKM/result_blast.html#bco:Bcell_0298) 1e-05

[crn:CAR_c08130](http://www.genome.jp/dbget-bin/www_bget?crn:CAR_c08130) cspA; cold shock protein CspA; K03704 cold shock ... [52](http://www.genome.jp/tmp/blast/120120152928c0IKM/result_blast.html#crn:CAR_c08130) 2e-05

[has:Halsa_2152](http://www.genome.jp/dbget-bin/www_bget?has:Halsa_2152) cold-shock protein; K03704 cold shock protein (be... [51](http://www.genome.jp/tmp/blast/120120152928c0IKM/result_blast.html#has:Halsa_2152) 2e-05

[pjd:Pjdr2_5748](http://www.genome.jp/dbget-bin/www_bget?pjd:Pjdr2_5748) cold-shock protein; K03704 cold shock protein (be... [51](http://www.genome.jp/tmp/blast/120120152928c0IKM/result_blast.html#pjd:Pjdr2_5748) 3e-05

[gym:GYMC10_4615](http://www.genome.jp/dbget-bin/www_bget?gym:GYMC10_4615) cold-shock DNA-binding domain protein; K03704 co... [51](http://www.genome.jp/tmp/blast/120120152928c0IKM/result_blast.html#gym:GYMC10_4615) 3e-05

[gym:GYMC10_5860](http://www.genome.jp/dbget-bin/www_bget?gym:GYMC10_5860) cold-shock DNA-binding domain protein; K03704 co... [51](http://www.genome.jp/tmp/blast/120120152928c0IKM/result_blast.html#gym:GYMC10_5860) 3e-05

[afl:Aflv_1485](http://www.genome.jp/dbget-bin/www_bget?afl:Aflv_1485) cspD; cold shock protein, CspA family; K03704 cold... [51](http://www.genome.jp/tmp/blast/120120152928c0IKM/result_blast.html#afl:Aflv_1485) 3e-05

[hor:Hore_05490](http://www.genome.jp/dbget-bin/www_bget?hor:Hore_05490) putative cold-shock DNA-binding domain-containing... [50](http://www.genome.jp/tmp/blast/120120152928c0IKM/result_blast.html#hor:Hore_05490) 5e-05

[oih:OB0579](http://www.genome.jp/dbget-bin/www_bget?oih:OB0579) cspC; cold shock protein; K03704 cold shock protein (... [50](http://www.genome.jp/tmp/blast/120120152928c0IKM/result_blast.html#oih:OB0579) 5e-05

[bse:Bsel_0503](http://www.genome.jp/dbget-bin/www_bget?bse:Bsel_0503) cold-shock DNA-binding domain-containing protein; ... [50](http://www.genome.jp/tmp/blast/120120152928c0IKM/result_blast.html#bse:Bsel_0503) 5e-05

[bcq:BCQ_5012](http://www.genome.jp/dbget-bin/www_bget?bcq:BCQ_5012) cspC; cold shock protein cspc; K03704 cold shock pr... [50](http://www.genome.jp/tmp/blast/120120152928c0IKM/result_blast.html#bcq:BCQ_5012) 6e-05

[bcr:BCAH187_A5356](http://www.genome.jp/dbget-bin/www_bget?bcr:BCAH187_A5356) cspC; cold shock protein CspC; K03704 cold sho... [50](http://www.genome.jp/tmp/blast/120120152928c0IKM/result_blast.html#bcr:BCAH187_A5356) 6e-05

[bca:BCE_5298](http://www.genome.jp/dbget-bin/www_bget?bca:BCE_5298) cspC; cold shock protein CspC; K03704 cold shock pr... [50](http://www.genome.jp/tmp/blast/120120152928c0IKM/result_blast.html#bca:BCE_5298) 6e-05

[ppm:PPSC2_c1893](http://www.genome.jp/dbget-bin/www_bget?ppm:PPSC2_c1893) cold shock cspb; K03704 cold shock protein (beta... [50](http://www.genome.jp/tmp/blast/120120152928c0IKM/result_blast.html#ppm:PPSC2_c1893) 7e-05

[chy:CHY_0155](http://www.genome.jp/dbget-bin/www_bget?chy:CHY_0155) cspC1; cold shock protein CspC; K03704 cold shock p... [50](http://www.genome.jp/tmp/blast/120120152928c0IKM/result_blast.html#chy:CHY_0155) 7e-05

[pms:KNP414_00115](http://www.genome.jp/dbget-bin/www_bget?pms:KNP414_00115) cspC; CspC; K03704 cold shock protein (beta-rib... [50](http://www.genome.jp/tmp/blast/120120152928c0IKM/result_blast.html#pms:KNP414_00115) 7e-05

[eat:EAT1b_0898](http://www.genome.jp/dbget-bin/www_bget?eat:EAT1b_0898) cold-shock protein; K03704 cold shock protein (be... [50](http://www.genome.jp/tmp/blast/120120152928c0IKM/result_blast.html#eat:EAT1b_0898) 7e-05

[bmd:BMD_1450](http://www.genome.jp/dbget-bin/www_bget?bmd:BMD_1450) cspD; cold shock protein; K03704 cold shock protein... [50](http://www.genome.jp/tmp/blast/120120152928c0IKM/result_blast.html#bmd:BMD_1450) 7e-05

[bmq:BMQ_1468](http://www.genome.jp/dbget-bin/www_bget?bmq:BMQ_1468) cspD; cold shock protein; K03704 cold shock protein... [50](http://www.genome.jp/tmp/blast/120120152928c0IKM/result_blast.html#bmq:BMQ_1468) 7e-05

[bpf:BpOF4_05540](http://www.genome.jp/dbget-bin/www_bget?bpf:BpOF4_05540) cspC; cold shock protein CspC; K03704 cold shock... [50](http://www.genome.jp/tmp/blast/120120152928c0IKM/result_blast.html#bpf:BpOF4_05540) 8e-05

[hor:Hore_05400](http://www.genome.jp/dbget-bin/www_bget?hor:Hore_05400) putative cold-shock DNA-binding domain-containing... [50](http://www.genome.jp/tmp/blast/120120152928c0IKM/result_blast.html#hor:Hore_05400) 8e-05

[bag:Bcoa_3297](http://www.genome.jp/dbget-bin/www_bget?bag:Bcoa_3297) cold-shock DNA-binding domain-containing protein; ... [50](http://www.genome.jp/tmp/blast/120120152928c0IKM/result_blast.html#bag:Bcoa_3297) 8e-05

[bck:BCO26_1235](http://www.genome.jp/dbget-bin/www_bget?bck:BCO26_1235) cspD; cold-shock DNA-binding domain-containing pr... [50](http://www.genome.jp/tmp/blast/120120152928c0IKM/result_blast.html#bck:BCO26_1235) 8e-05

[sab:SAB0749](http://www.genome.jp/dbget-bin/www_bget?sab:SAB0749) cspC; cold-shock protein C; K03704 cold shock protei... [49](http://www.genome.jp/tmp/blast/120120152928c0IKM/result_blast.html#sab:SAB0749) 8e-05

[sad:SAAV_0762](http://www.genome.jp/dbget-bin/www_bget?sad:SAAV_0762) CSD family cold shock protein; K03704 cold shock p... [49](http://www.genome.jp/tmp/blast/120120152928c0IKM/result_blast.html#sad:SAAV_0762) 8e-05

[sae:NWMN_0761](http://www.genome.jp/dbget-bin/www_bget?sae:NWMN_0761) cspC; cold-shock protein CSD family protein; K0370... [49](http://www.genome.jp/tmp/blast/120120152928c0IKM/result_blast.html#sae:NWMN_0761) 8e-05

[sao:SAOUHSC_00819](http://www.genome.jp/dbget-bin/www_bget?sao:SAOUHSC_00819) hypothetical protein; K03704 cold shock protei... [49](http://www.genome.jp/tmp/blast/120120152928c0IKM/result_blast.html#sao:SAOUHSC_00819) 8e-05

[saa:SAUSA300_0777](http://www.genome.jp/dbget-bin/www_bget?saa:SAUSA300_0777) cold shock protein; K03704 cold shock protein ... [49](http://www.genome.jp/tmp/blast/120120152928c0IKM/result_blast.html#saa:SAUSA300_0777) 8e-05

[sax:USA300HOU_0824](http://www.genome.jp/dbget-bin/www_bget?sax:USA300HOU_0824) csp; cold-shock protein; K03704 cold shock pr... [49](http://www.genome.jp/tmp/blast/120120152928c0IKM/result_blast.html#sax:USA300HOU_0824) 8e-05

[sac:SACOL0861](http://www.genome.jp/dbget-bin/www_bget?sac:SACOL0861) CSD family cold shock protein; K03704 cold shock p... [49](http://www.genome.jp/tmp/blast/120120152928c0IKM/result_blast.html#sac:SACOL0861) 8e-05

[sar:SAR0848](http://www.genome.jp/dbget-bin/www_bget?sar:SAR0848) cspC; cold shock protein; K03704 cold shock protein ... [49](http://www.genome.jp/tmp/blast/120120152928c0IKM/result_blast.html#sar:SAR0848) 8e-05

[sas:SAS0757](http://www.genome.jp/dbget-bin/www_bget?sas:SAS0757) putative cold shock protein; K03704 cold shock prote... [49](http://www.genome.jp/tmp/blast/120120152928c0IKM/result_blast.html#sas:SAS0757) 8e-05

[sam:MW0770](http://www.genome.jp/dbget-bin/www_bget?sam:MW0770) cspC; cold-shock protein C; K03704 cold shock protein... [49](http://www.genome.jp/tmp/blast/120120152928c0IKM/result_blast.html#sam:MW0770) 8e-05

[saj:SaurJH9_0817](http://www.genome.jp/dbget-bin/www_bget?saj:SaurJH9_0817) cold-shock DNA-binding domain-containing protei... [49](http://www.genome.jp/tmp/blast/120120152928c0IKM/result_blast.html#saj:SaurJH9_0817) 8e-05

[sah:SaurJH1_0833](http://www.genome.jp/dbget-bin/www_bget?sah:SaurJH1_0833) DNA-binding cold-shock protein; K03704 cold sho... [49](http://www.genome.jp/tmp/blast/120120152928c0IKM/result_blast.html#sah:SaurJH1_0833) 8e-05

[saw:SAHV_0812](http://www.genome.jp/dbget-bin/www_bget?saw:SAHV_0812) cspC; cold-shock protein C; K03704 cold shock prot... [49](http://www.genome.jp/tmp/blast/120120152928c0IKM/result_blast.html#saw:SAHV_0812) 8e-05

[sav:SAV0816](http://www.genome.jp/dbget-bin/www_bget?sav:SAV0816) cspC; cold-shock protein C; K03704 cold shock protei... [49](http://www.genome.jp/tmp/blast/120120152928c0IKM/result_blast.html#sav:SAV0816) 8e-05

[sau:SA0747](http://www.genome.jp/dbget-bin/www_bget?sau:SA0747) cspC; cold-shock protein C; K03704 cold shock protein... [49](http://www.genome.jp/tmp/blast/120120152928c0IKM/result_blast.html#sau:SA0747) 8e-05

[bae:BATR1942_08815](http://www.genome.jp/dbget-bin/www_bget?bae:BATR1942_08815) RNA-helicase co-factor; K03704 cold shock pro... [49](http://www.genome.jp/tmp/blast/120120152928c0IKM/result_blast.html#bae:BATR1942_08815) 9e-05

[bao:BAMF_2093](http://www.genome.jp/dbget-bin/www_bget?bao:BAMF_2093) cspD; cold-shock protein, molecular chaperone, RNA... [49](http://www.genome.jp/tmp/blast/120120152928c0IKM/result_blast.html#bao:BAMF_2093) 9e-05

[bay:RBAM_020070](http://www.genome.jp/dbget-bin/www_bget?bay:RBAM_020070) cspD; hypothetical protein; K03704 cold shock pr... [49](http://www.genome.jp/tmp/blast/120120152928c0IKM/result_blast.html#bay:RBAM_020070) 9e-05

[bld:BLi02332](http://www.genome.jp/dbget-bin/www_bget?bld:BLi02332) cspD; CspD; K03704 cold shock protein (beta-ribbon,... [49](http://www.genome.jp/tmp/blast/120120152928c0IKM/result_blast.html#bld:BLi02332) 9e-05

[bli:BL05226](http://www.genome.jp/dbget-bin/www_bget?bli:BL05226) cspD; cold-shock protein; K03704 cold shock protein ... [49](http://www.genome.jp/tmp/blast/120120152928c0IKM/result_blast.html#bli:BL05226) 9e-05

[bsn:BSn5_01425](http://www.genome.jp/dbget-bin/www_bget?bsn:BSn5_01425) cold-shock protein, molecular chaperone, RNA-heli... [49](http://www.genome.jp/tmp/blast/120120152928c0IKM/result_blast.html#bsn:BSn5_01425) 9e-05

[bst:GYO_2417](http://www.genome.jp/dbget-bin/www_bget?bst:GYO_2417) cold shock protein cspD; K03704 cold shock protein ... [49](http://www.genome.jp/tmp/blast/120120152928c0IKM/result_blast.html#bst:GYO_2417) 9e-05

[bss:BSUW23_10735](http://www.genome.jp/dbget-bin/www_bget?bss:BSUW23_10735) cspD; RNA-helicase co-factor; K03704 cold shock... [49](http://www.genome.jp/tmp/blast/120120152928c0IKM/result_blast.html#bss:BSUW23_10735) 9e-05

[bsu:BSU21930](http://www.genome.jp/dbget-bin/www_bget?bsu:BSU21930) cspD; cold-shock protein; K03704 cold shock protein... [49](http://www.genome.jp/tmp/blast/120120152928c0IKM/result_blast.html#bsu:BSU21930) 9e-05

[bcy:Bcer98_1327](http://www.genome.jp/dbget-bin/www_bget?bcy:Bcer98_1327) cold-shock DNA-binding domain-containing protein... [49](http://www.genome.jp/tmp/blast/120120152928c0IKM/result_blast.html#bcy:Bcer98_1327) 9e-05

[cno:NT01CX_0617](http://www.genome.jp/dbget-bin/www_bget?cno:NT01CX_0617) cspB; cold shock protein cspB; K03704 cold shock... [49](http://www.genome.jp/tmp/blast/120120152928c0IKM/result_blast.html#cno:NT01CX_0617) 9e-05

[ain:Acin_2369](http://www.genome.jp/dbget-bin/www_bget?ain:Acin_2369) cold-shock DNA-binding protein; K03704 cold shock ... [49](http://www.genome.jp/tmp/blast/120120152928c0IKM/result_blast.html#ain:Acin_2369) 9e-05

[afn:Acfer_0196](http://www.genome.jp/dbget-bin/www_bget?afn:Acfer_0196) cold-shock protein; K03704 cold shock protein (be... [49](http://www.genome.jp/tmp/blast/120120152928c0IKM/result_blast.html#afn:Acfer_0196) 9e-05

[cbn:CbC4_2353](http://www.genome.jp/dbget-bin/www_bget?cbn:CbC4_2353) major cold shock protein; K03704 cold shock protei... [49](http://www.genome.jp/tmp/blast/120120152928c0IKM/result_blast.html#cbn:CbC4_2353) 1e-04

[pms:KNP414_01584](http://www.genome.jp/dbget-bin/www_bget?pms:KNP414_01584) cspB; CspB; K03704 cold shock protein (beta-rib... [49](http://www.genome.jp/tmp/blast/120120152928c0IKM/result_blast.html#pms:KNP414_01584) 1e-04

[bwe:BcerKBAB4_4985](http://www.genome.jp/dbget-bin/www_bget?bwe:BcerKBAB4_4985) cold-shock DNA-binding domain-containing prot... [49](http://www.genome.jp/tmp/blast/120120152928c0IKM/result_blast.html#bwe:BcerKBAB4_4985) 1e-04

[btb:BMB171_C4782](http://www.genome.jp/dbget-bin/www_bget?btb:BMB171_C4782) cspC; cold shock protein; K03704 cold shock pro... [49](http://www.genome.jp/tmp/blast/120120152928c0IKM/result_blast.html#btb:BMB171_C4782) 1e-04

[btl:BALH_4686](http://www.genome.jp/dbget-bin/www_bget?btl:BALH_4686) cspC; cold-shock DNA-binding protein family protei... [49](http://www.genome.jp/tmp/blast/120120152928c0IKM/result_blast.html#btl:BALH_4686) 1e-04

[btk:BT9727_4870](http://www.genome.jp/dbget-bin/www_bget?btk:BT9727_4870) cspC; cold shock protein; K03704 cold shock prot... [49](http://www.genome.jp/tmp/blast/120120152928c0IKM/result_blast.html#btk:BT9727_4870) 1e-04

[bcx:BCA_5321](http://www.genome.jp/dbget-bin/www_bget?bcx:BCA_5321) cspC; cold shock protein CspC; K03704 cold shock pr... [49](http://www.genome.jp/tmp/blast/120120152928c0IKM/result_blast.html#bcx:BCA_5321) 1e-04

[bcu:BCAH820_5280](http://www.genome.jp/dbget-bin/www_bget?bcu:BCAH820_5280) cspC; cold shock protein CspC; K03704 cold shoc... [49](http://www.genome.jp/tmp/blast/120120152928c0IKM/result_blast.html#bcu:BCAH820_5280) 1e-04

[bcz:BCZK4885](http://www.genome.jp/dbget-bin/www_bget?bcz:BCZK4885) cspC; cold shock protein; K03704 cold shock protein... [49](http://www.genome.jp/tmp/blast/120120152928c0IKM/result_blast.html#bcz:BCZK4885) 1e-04

[bce:BC5191](http://www.genome.jp/dbget-bin/www_bget?bce:BC5191) cold shock protein; K03704 cold shock protein (beta-r... [49](http://www.genome.jp/tmp/blast/120120152928c0IKM/result_blast.html#bce:BC5191) 1e-04

[bai:BAA_5453](http://www.genome.jp/dbget-bin/www_bget?bai:BAA_5453) cspC; cold shock protein CspC; K03704 cold shock pr... [49](http://www.genome.jp/tmp/blast/120120152928c0IKM/result_blast.html#bai:BAA_5453) 1e-04

[bat:BAS5040](http://www.genome.jp/dbget-bin/www_bget?bat:BAS5040) cold shock protein CspC; K03704 cold shock protein (... [49](http://www.genome.jp/tmp/blast/120120152928c0IKM/result_blast.html#bat:BAS5040) 1e-04

[hor:Hore_16470](http://www.genome.jp/dbget-bin/www_bget?hor:Hore_16470) putative cold-shock DNA-binding domain-containing... [49](http://www.genome.jp/tmp/blast/120120152928c0IKM/result_blast.html#hor:Hore_16470) 1e-04

[bal:BACI_c51770](http://www.genome.jp/dbget-bin/www_bget?bal:BACI_c51770) cspC; cold shock protein; K03704 cold shock prot... [49](http://www.genome.jp/tmp/blast/120120152928c0IKM/result_blast.html#bal:BACI_c51770) 1e-04

[nth:Nther_1584](http://www.genome.jp/dbget-bin/www_bget?nth:Nther_1584) cold-shock DNA-binding protein family; K03704 col... [49](http://www.genome.jp/tmp/blast/120120152928c0IKM/result_blast.html#nth:Nther_1584) 1e-04

[bcg:BCG9842_B5647](http://www.genome.jp/dbget-bin/www_bget?bcg:BCG9842_B5647) cspC; cold-shock protein CspC; K03704 cold sho... [49](http://www.genome.jp/tmp/blast/120120152928c0IKM/result_blast.html#bcg:BCG9842_B5647) 1e-04

[bcb:BCB4264_A5310](http://www.genome.jp/dbget-bin/www_bget?bcb:BCB4264_A5310) cspC; cold shock protein CspC; K03704 cold sho... [49](http://www.genome.jp/tmp/blast/120120152928c0IKM/result_blast.html#bcb:BCB4264_A5310) 1e-04

[bah:BAMEG_5475](http://www.genome.jp/dbget-bin/www_bget?bah:BAMEG_5475) cspC; cold shock protein CspC; K03704 cold shock ... [49](http://www.genome.jp/tmp/blast/120120152928c0IKM/result_blast.html#bah:BAMEG_5475) 1e-04

[bar:GBAA_5424](http://www.genome.jp/dbget-bin/www_bget?bar:GBAA_5424) cspC; cold shock protein CspC; K03704 cold shock p... [49](http://www.genome.jp/tmp/blast/120120152928c0IKM/result_blast.html#bar:GBAA_5424) 1e-04

[ban:BA_5424](http://www.genome.jp/dbget-bin/www_bget?ban:BA_5424) cspC; cold shock protein CspC; K03704 cold shock pro... [49](http://www.genome.jp/tmp/blast/120120152928c0IKM/result_blast.html#ban:BA_5424) 1e-04

[bcl:ABC3070](http://www.genome.jp/dbget-bin/www_bget?bcl:ABC3070) cold shock protein CspC; K03704 cold shock protein (... [49](http://www.genome.jp/tmp/blast/120120152928c0IKM/result_blast.html#bcl:ABC3070) 1e-04

[nth:Nther_2223](http://www.genome.jp/dbget-bin/www_bget?nth:Nther_2223) cold-shock DNA-binding protein family; K03704 col... [49](http://www.genome.jp/tmp/blast/120120152928c0IKM/result_blast.html#nth:Nther_2223) 1e-04

[bco:Bcell_3592](http://www.genome.jp/dbget-bin/www_bget?bco:Bcell_3592) cold-shock DNA-binding domain-containing protein;... [49](http://www.genome.jp/tmp/blast/120120152928c0IKM/result_blast.html#bco:Bcell_3592) 1e-04

Bottom of Form

[DBGET](http://www.genome.jp/dbget/) integrated database retrieval system, [GenomeNet](http://www.genome.jp/)

(Out of the 500 hits obtained the 100 hits are shown above). The list includes some organisms in the same genera)

2. Blast search of KEGG genome database of *Halomonas elongata for homologue of 12 kDa protein*

## BLASTP Search Result *Halomonas eurihalina* 12 kDa cold shock protein

**Database: hel**

**Protein sequence database entries related to query** - 4 hits

Top of Form

command: blastp

query: /share/www/blast/120120152259HNXKC/query.seq

database: h.elongata.pep (prot)

Entry bits E-val

--------------------------------------------------------------------- -----------

[hel:HELO_1644](http://www.genome.jp/dbget-bin/www_bget?hel:HELO_1644) csp; cold shock protein CspA; K03704 cold shock pr... [36](http://www.genome.jp/tmp/blast/120120152259HNXKC/result_blast.html#hel:HELO_1644) 5e-04

[hel:HELO_3431](http://www.genome.jp/dbget-bin/www_bget?hel:HELO_3431) csp; cold shock protein CspA; K03704 cold shock pr... [35](http://www.genome.jp/tmp/blast/120120152259HNXKC/result_blast.html#hel:HELO_3431) 0.001

[hel:HELO_3240](http://www.genome.jp/dbget-bin/www_bget?hel:HELO_3240) csp; cold shock protein CspA; K03704 cold shock pr... [33](http://www.genome.jp/tmp/blast/120120152259HNXKC/result_blast.html#hel:HELO_3240) 0.003

[hel:HELO_3812](http://www.genome.jp/dbget-bin/www_bget?hel:HELO_3812) cold shock protein CspA; K03704 cold shock protein... [26](http://www.genome.jp/tmp/blast/120120152259HNXKC/result_blast.html#hel:HELO_3812) 0.43

Bottom of Form

[DBGET](http://www.genome.jp/dbget/) integrated database retrieval system, [GenomeNet](http://www.genome.jp/)

3. ClustalW2 Results

CLUSTAL 2.1 multiple sequence alignment 12 kDa cold shock protein (query)

**hel_HELO_3431 ------------------------------------------------------------**

**hel_HELO_3240 ------------------------------------------------------------**

**hel_HELO_1644 ------------------------------------------------------------**

**query ------------------------------------------------------------**

**hel_HELO_3812 MNRKVVLRCSLISLLLAAPSPLLVALFVHLAGGAMSREIFASLEIGGVGAVYLAVALAVF 60**

**hel_HELO_3431 ---------------------------MATGTVKWFNDTKGYGFISPEDGGDDLFVHFSE 33**

**hel_HELO_3240 ---------------------------MATGTVKWFNDTKGFGFISPDDNGDDLFAHFSE 33**

**hel_HELO_1644 ---------------------------MTTGTVKWFNDSKGFGFISPADGGDDVFAHFSE 33**

**query ------------------------------------------------------------**

**hel_HELO_3812 VLLLIATLAVNALTPQLVNLAEVEDDDREIGEVKWFNVNKGYGFITR-DSGEDVFVHFRA 119**

**hel_HELO_3431 IQAEGFKSLQDGQKVSFEVTQGKKGLQASNVRVTD 68**

**hel_HELO_3240 IQAEGFKSLQDGQKVSFDVTQGKKGLQASNIKVVD 68**

**hel_HELO_1644 IQAEGFKSLQEGQSVSFDVTQGKKGLQASNIKALG 68**

**query ------KSLEEGQAVEFEVVEGDRGPQAANVVKL- 28**

**hel_HELO_3812 IRGRGHRTLAEGQKVKYQVSRNERGLQADDVTVIT 154**

**::* :** *.::* ...:* ** ::**


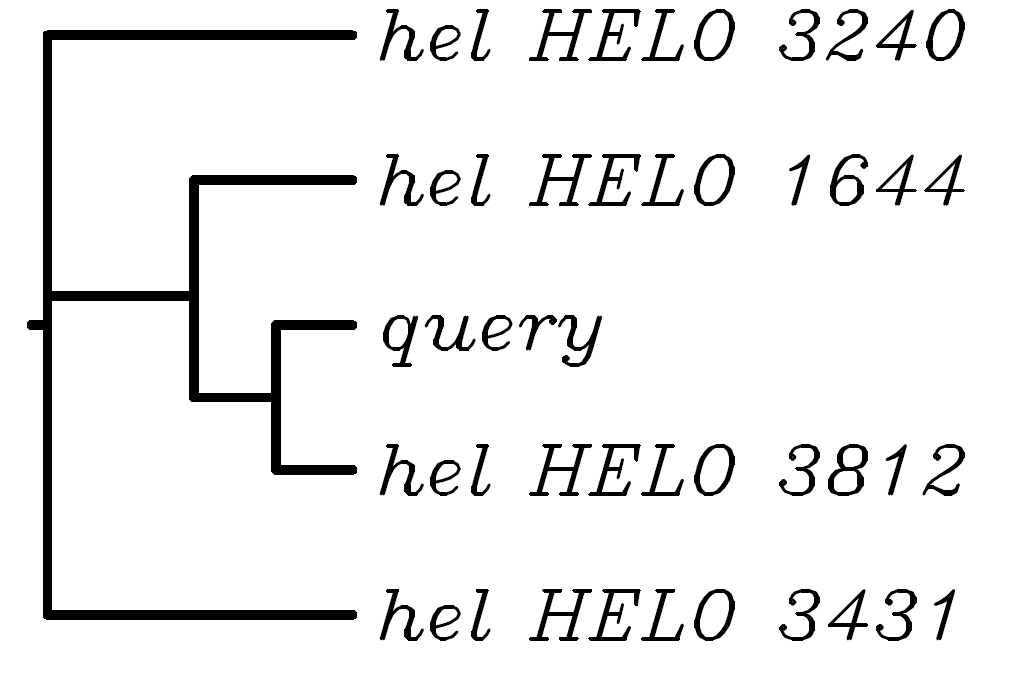


## Rooted phylogenetic tree (neighbor joining) of 12 kDa protein showing similarity to HELO_3812

## HELO_3431, HELO_3240, HELO_1644 are 68 amino acid residue cold shock proteins of CspA family, HELO_3812 is158 amino acid residue cold shock protein of CspA family

## 4. Blast search of KEGG genome database

## BLASTP Search Result *Halomonas eurihalina* 8 kDa cold shock protein

**Database: genes**

**Protein sequence database entries related to query** - 202 hits

Top of Form

command: blastp

query: /share/www/blast/120120152646PUrad/query.seq

database: genes (prot)

------------------------

command query database

blastp prot prot

Entry bits E-val

--------------------------------------------------------------------- -----------

[pin:Ping_1881](http://www.genome.jp/dbget-bin/www_bget?pin:Ping_1881) cold-shock DNA-binding domain-containing protein; ... [39](http://www.genome.jp/tmp/blast/120120152646PUrad/result_blast.html#pin:Ping_1881) 0.091

[pin:Ping_0279](http://www.genome.jp/dbget-bin/www_bget?pin:Ping_0279) cold-shock DNA-binding domain-containing protein [39](http://www.genome.jp/tmp/blast/120120152646PUrad/result_blast.html#pin:Ping_0279) 0.11

[gag:Glaag_2787](http://www.genome.jp/dbget-bin/www_bget?gag:Glaag_2787) cold-shock DNA-binding domain protein; K03704 col... [39](http://www.genome.jp/tmp/blast/120120152646PUrad/result_blast.html#gag:Glaag_2787) 0.13

[pat:Patl_1543](http://www.genome.jp/dbget-bin/www_bget?pat:Patl_1543) cold-shock DNA-binding domain-containing protein; ... [39](http://www.genome.jp/tmp/blast/120120152646PUrad/result_blast.html#pat:Patl_1543) 0.13

[pat:Patl_3784](http://www.genome.jp/dbget-bin/www_bget?pat:Patl_3784) cold-shock DNA-binding domain-containing protein; ... [38](http://www.genome.jp/tmp/blast/120120152646PUrad/result_blast.html#pat:Patl_3784) 0.18

[pin:Ping_2543](http://www.genome.jp/dbget-bin/www_bget?pin:Ping_2543) cold-shock DNA-binding domain-containing protein; ... [38](http://www.genome.jp/tmp/blast/120120152646PUrad/result_blast.html#pin:Ping_2543) 0.19

[pin:Ping_3704](http://www.genome.jp/dbget-bin/www_bget?pin:Ping_3704) cold-shock DNA-binding domain-containing protein; ... [38](http://www.genome.jp/tmp/blast/120120152646PUrad/result_blast.html#pin:Ping_3704) 0.19

[pin:Ping_3098](http://www.genome.jp/dbget-bin/www_bget?pin:Ping_3098) cold-shock DNA-binding domain-containing protein; ... [38](http://www.genome.jp/tmp/blast/120120152646PUrad/result_blast.html#pin:Ping_3098) 0.20

[sde:Sde_3558](http://www.genome.jp/dbget-bin/www_bget?sde:Sde_3558) cold-shock DNA-binding protein family protein; K037... [38](http://www.genome.jp/tmp/blast/120120152646PUrad/result_blast.html#sde:Sde_3558) 0.23

[sde:Sde_3760](http://www.genome.jp/dbget-bin/www_bget?sde:Sde_3760) cold-shock DNA-binding protein family protein; K037... [37](http://www.genome.jp/tmp/blast/120120152646PUrad/result_blast.html#sde:Sde_3760) 0.24

[pcr:Pcryo_2133](http://www.genome.jp/dbget-bin/www_bget?pcr:Pcryo_2133) cold-shock DNA-binding domain-containing protein;... [37](http://www.genome.jp/tmp/blast/120120152646PUrad/result_blast.html#pcr:Pcryo_2133) 0.28

[par:Psyc_1846](http://www.genome.jp/dbget-bin/www_bget?par:Psyc_1846) cold-shock DNA-binding protein family protein; K03... [37](http://www.genome.jp/tmp/blast/120120152646PUrad/result_blast.html#par:Psyc_1846) 0.28

[asa:ASA_0864](http://www.genome.jp/dbget-bin/www_bget?asa:ASA_0864) cspX; cold shock protein; K03704 cold shock protein... [37](http://www.genome.jp/tmp/blast/120120152646PUrad/result_blast.html#asa:ASA_0864) 0.31

[maq:Maqu_3612](http://www.genome.jp/dbget-bin/www_bget?maq:Maqu_3612) cold-shock DNA-binding domain-containing protein; ... [37](http://www.genome.jp/tmp/blast/120120152646PUrad/result_blast.html#maq:Maqu_3612) 0.31

[pin:Ping_2158](http://www.genome.jp/dbget-bin/www_bget?pin:Ping_2158) cold-shock DNA-binding domain-containing protein; ... [37](http://www.genome.jp/tmp/blast/120120152646PUrad/result_blast.html#pin:Ping_2158) 0.33

[maq:Maqu_1336](http://www.genome.jp/dbget-bin/www_bget?maq:Maqu_1336) cold-shock DNA-binding domain-containing protein; ... [37](http://www.genome.jp/tmp/blast/120120152646PUrad/result_blast.html#maq:Maqu_1336) 0.35

[cps:CPS_4529](http://www.genome.jp/dbget-bin/www_bget?cps:CPS_4529) cold shock DNA-binding domain-containing protein; K... [37](http://www.genome.jp/tmp/blast/120120152646PUrad/result_blast.html#cps:CPS_4529) 0.36

[pin:Ping_3095](http://www.genome.jp/dbget-bin/www_bget?pin:Ping_3095) cold-shock DNA-binding domain-containing protein; ... [37](http://www.genome.jp/tmp/blast/120120152646PUrad/result_blast.html#pin:Ping_3095) 0.38

[pha:PSHAa2980](http://www.genome.jp/dbget-bin/www_bget?pha:PSHAa2980) cspE; cold shock-like protein CspE, RNA chaperone,... [37](http://www.genome.jp/tmp/blast/120120152646PUrad/result_blast.html#pha:PSHAa2980) 0.41

[pha:PSHAa2979](http://www.genome.jp/dbget-bin/www_bget?pha:PSHAa2979) cspE; RNA chaperone, transcription antiterminator,... [37](http://www.genome.jp/tmp/blast/120120152646PUrad/result_blast.html#pha:PSHAa2979) 0.41

[pha:PSHAb0384](http://www.genome.jp/dbget-bin/www_bget?pha:PSHAb0384) cspX; cold shock protein; K03704 cold shock protei... [37](http://www.genome.jp/tmp/blast/120120152646PUrad/result_blast.html#pha:PSHAb0384) 0.41

[pha:PSHAb0387](http://www.genome.jp/dbget-bin/www_bget?pha:PSHAb0387) cspX; cold shock protein; K03704 cold shock protei... [37](http://www.genome.jp/tmp/blast/120120152646PUrad/result_blast.html#pha:PSHAb0387) 0.45

[psm:PSM_B0051](http://www.genome.jp/dbget-bin/www_bget?psm:PSM_B0051) cspX; cold shock protein; K03704 cold shock protei... [37](http://www.genome.jp/tmp/blast/120120152646PUrad/result_blast.html#psm:PSM_B0051) 0.47

[pha:PSHAb0386](http://www.genome.jp/dbget-bin/www_bget?pha:PSHAb0386) cspX; cold shock protein; K03704 cold shock protei... [37](http://www.genome.jp/tmp/blast/120120152646PUrad/result_blast.html#pha:PSHAb0386) 0.47

[pha:PSHAa2978](http://www.genome.jp/dbget-bin/www_bget?pha:PSHAa2978) cspE; RNA chaperone, transcription antiterminator,... [37](http://www.genome.jp/tmp/blast/120120152646PUrad/result_blast.html#pha:PSHAa2978) 0.47

[tau:Tola_1278](http://www.genome.jp/dbget-bin/www_bget?tau:Tola_1278) cold-shock DNA-binding domain-containing protein; ... [37](http://www.genome.jp/tmp/blast/120120152646PUrad/result_blast.html#tau:Tola_1278) 0.50

[hch:HCH_04707](http://www.genome.jp/dbget-bin/www_bget?hch:HCH_04707) cold shock protein; K03704 cold shock protein (bet... [37](http://www.genome.jp/tmp/blast/120120152646PUrad/result_blast.html#hch:HCH_04707) 0.50

[psm:PSM_B0081](http://www.genome.jp/dbget-bin/www_bget?psm:PSM_B0081) cspX; cold shock protein; K03704 cold shock protei... [36](http://www.genome.jp/tmp/blast/120120152646PUrad/result_blast.html#psm:PSM_B0081) 0.64

[vsp:VS_II0495](http://www.genome.jp/dbget-bin/www_bget?vsp:VS_II0495) cold-shock protein; K03704 cold shock protein (bet... [36](http://www.genome.jp/tmp/blast/120120152646PUrad/result_blast.html#vsp:VS_II0495) 0.64

[pha:PSHAb0078](http://www.genome.jp/dbget-bin/www_bget?pha:PSHAb0078) cspX; cold shock protein; K03704 cold shock protei... [36](http://www.genome.jp/tmp/blast/120120152646PUrad/result_blast.html#pha:PSHAb0078) 0.66

[maq:Maqu_2955](http://www.genome.jp/dbget-bin/www_bget?maq:Maqu_2955) cold-shock DNA-binding domain-containing protein; ... [36](http://www.genome.jp/tmp/blast/120120152646PUrad/result_blast.html#maq:Maqu_2955) 0.72

[pin:Ping_1953](http://www.genome.jp/dbget-bin/www_bget?pin:Ping_1953) cold-shock DNA-binding domain-containing protein; ... [36](http://www.genome.jp/tmp/blast/120120152646PUrad/result_blast.html#pin:Ping_1953) 0.79

[vcj:VCD_000065](http://www.genome.jp/dbget-bin/www_bget?vcj:VCD_000065) cold shock protein CspE; K03704 cold shock protei... [36](http://www.genome.jp/tmp/blast/120120152646PUrad/result_blast.html#vcj:VCD_000065) 0.84

[vcm:VCM66_A0180](http://www.genome.jp/dbget-bin/www_bget?vcm:VCM66_A0180) cold shock DNA-binding domain protein; K03704 co... [36](http://www.genome.jp/tmp/blast/120120152646PUrad/result_blast.html#vcm:VCM66_A0180) 0.84

[vco:VC0395_1092](http://www.genome.jp/dbget-bin/www_bget?vco:VC0395_1092) cold shock DNA-binding domain-containing protein... [36](http://www.genome.jp/tmp/blast/120120152646PUrad/result_blast.html#vco:VC0395_1092) 0.84

[vce:Vch1786_II0979](http://www.genome.jp/dbget-bin/www_bget?vce:Vch1786_II0979) cold shock protein (beta-ribbon, CspA family)... [36](http://www.genome.jp/tmp/blast/120120152646PUrad/result_blast.html#vce:Vch1786_II0979) 0.84

[vch:VCA0184](http://www.genome.jp/dbget-bin/www_bget?vch:VCA0184) cold shock DNA-binding domain-containing protein; K0... [36](http://www.genome.jp/tmp/blast/120120152646PUrad/result_blast.html#vch:VCA0184) 0.84

[aha:AHA_3450](http://www.genome.jp/dbget-bin/www_bget?aha:AHA_3450) hypothetical protein; K03704 cold shock protein (be... [35](http://www.genome.jp/tmp/blast/120120152646PUrad/result_blast.html#aha:AHA_3450) 0.93

[avr:B565_3280](http://www.genome.jp/dbget-bin/www_bget?avr:B565_3280) cold shock-like protein CspE; K03704 cold shock pr... [35](http://www.genome.jp/tmp/blast/120120152646PUrad/result_blast.html#avr:B565_3280) 0.95

[pin:Ping_0755](http://www.genome.jp/dbget-bin/www_bget?pin:Ping_0755) cold-shock DNA-binding domain-containing protein; ... [35](http://www.genome.jp/tmp/blast/120120152646PUrad/result_blast.html#pin:Ping_0755) 0.99

[prw:PsycPRwf_2170](http://www.genome.jp/dbget-bin/www_bget?prw:PsycPRwf_2170) cold-shock DNA-binding domain-containing protein [35](http://www.genome.jp/tmp/blast/120120152646PUrad/result_blast.html#prw:PsycPRwf_2170) 1.0

[prw:PsycPRwf_0333](http://www.genome.jp/dbget-bin/www_bget?prw:PsycPRwf_0333) cold-shock DNA-binding domain-containing prote... [35](http://www.genome.jp/tmp/blast/120120152646PUrad/result_blast.html#prw:PsycPRwf_0333) 1.0

[acd:AOLE_05110](http://www.genome.jp/dbget-bin/www_bget?acd:AOLE_05110) cold shock protein; K03704 cold shock protein (be... [35](http://www.genome.jp/tmp/blast/120120152646PUrad/result_blast.html#acd:AOLE_05110) 1.0

[tcx:Tcr_1057](http://www.genome.jp/dbget-bin/www_bget?tcx:Tcr_1057) cold-shock DNA-binding domain-containing protein; K... [35](http://www.genome.jp/tmp/blast/120120152646PUrad/result_blast.html#tcx:Tcr_1057) 1.1

[sse:Ssed_2614](http://www.genome.jp/dbget-bin/www_bget?sse:Ssed_2614) cold-shock DNA-binding domain-containing protein; ... [35](http://www.genome.jp/tmp/blast/120120152646PUrad/result_blast.html#sse:Ssed_2614) 1.2

[slo:Shew_1614](http://www.genome.jp/dbget-bin/www_bget?slo:Shew_1614) cold-shock DNA-binding domain-containing protein; ... [35](http://www.genome.jp/tmp/blast/120120152646PUrad/result_blast.html#slo:Shew_1614) 1.2

[swp:swp_3032](http://www.genome.jp/dbget-bin/www_bget?swp:swp_3032) DNA-binding cold shock protein; K03704 cold shock p... [35](http://www.genome.jp/tmp/blast/120120152646PUrad/result_blast.html#swp:swp_3032) 1.3

[swd:Swoo_2052](http://www.genome.jp/dbget-bin/www_bget?swd:Swoo_2052) cold-shock DNA-binding domain-containing protein; ... [35](http://www.genome.jp/tmp/blast/120120152646PUrad/result_blast.html#swd:Swoo_2052) 1.3

[shl:Shal_1797](http://www.genome.jp/dbget-bin/www_bget?shl:Shal_1797) cold-shock DNA-binding domain-containing protein; ... [35](http://www.genome.jp/tmp/blast/120120152646PUrad/result_blast.html#shl:Shal_1797) 1.3

[spl:Spea_2485](http://www.genome.jp/dbget-bin/www_bget?spl:Spea_2485) cold-shock DNA-binding domain-containing protein; ... [35](http://www.genome.jp/tmp/blast/120120152646PUrad/result_blast.html#spl:Spea_2485) 1.3

[svo:SVI_2530](http://www.genome.jp/dbget-bin/www_bget?svo:SVI_2530) cold shock domain family protein; K03704 cold shock... [35](http://www.genome.jp/tmp/blast/120120152646PUrad/result_blast.html#svo:SVI_2530) 1.3

[asa:ASA_1471](http://www.genome.jp/dbget-bin/www_bget?asa:ASA_1471) cspX; cold shock protein; K03704 cold shock protein... [35](http://www.genome.jp/tmp/blast/120120152646PUrad/result_blast.html#asa:ASA_1471) 1.4

[aha:AHA_2864](http://www.genome.jp/dbget-bin/www_bget?aha:AHA_2864) hypothetical protein; K03704 cold shock protein (be... [35](http://www.genome.jp/tmp/blast/120120152646PUrad/result_blast.html#aha:AHA_2864) 1.4

[abo:ABO_0153](http://www.genome.jp/dbget-bin/www_bget?abo:ABO_0153) cspE; cold-shock protein; K03704 cold shock protein... [35](http://www.genome.jp/tmp/blast/120120152646PUrad/result_blast.html#abo:ABO_0153) 1.5

[sfr:Sfri_1693](http://www.genome.jp/dbget-bin/www_bget?sfr:Sfri_1693) cold-shock DNA-binding domain-containing protein; ... [35](http://www.genome.jp/tmp/blast/120120152646PUrad/result_blast.html#sfr:Sfri_1693) 1.5

[sdn:Sden_2285](http://www.genome.jp/dbget-bin/www_bget?sdn:Sden_2285) cold-shock protein, DNA-binding; K03704 cold shock... [35](http://www.genome.jp/tmp/blast/120120152646PUrad/result_blast.html#sdn:Sden_2285) 1.6

[shw:Sputw3181_2450](http://www.genome.jp/dbget-bin/www_bget?shw:Sputw3181_2450) cold-shock DNA-binding domain-containing prot... [35](http://www.genome.jp/tmp/blast/120120152646PUrad/result_blast.html#shw:Sputw3181_2450) 1.7

[spc:Sputcn32_1572](http://www.genome.jp/dbget-bin/www_bget?spc:Sputcn32_1572) cold-shock DNA-binding domain-containing prote... [35](http://www.genome.jp/tmp/blast/120120152646PUrad/result_blast.html#spc:Sputcn32_1572) 1.7

[shn:Shewana3_2569](http://www.genome.jp/dbget-bin/www_bget?shn:Shewana3_2569) cold-shock DNA-binding protein family protein;... [35](http://www.genome.jp/tmp/blast/120120152646PUrad/result_blast.html#shn:Shewana3_2569) 1.7

[shm:Shewmr7_2477](http://www.genome.jp/dbget-bin/www_bget?shm:Shewmr7_2477) cold-shock DNA-binding protein family protein; ... [35](http://www.genome.jp/tmp/blast/120120152646PUrad/result_blast.html#shm:Shewmr7_2477) 1.7

[she:Shewmr4_2407](http://www.genome.jp/dbget-bin/www_bget?she:Shewmr4_2407) cold-shock DNA-binding protein family protein; ... [35](http://www.genome.jp/tmp/blast/120120152646PUrad/result_blast.html#she:Shewmr4_2407) 1.7

[sbp:Sbal223_2659](http://www.genome.jp/dbget-bin/www_bget?sbp:Sbal223_2659) cold-shock DNA-binding domain-containing protei... [35](http://www.genome.jp/tmp/blast/120120152646PUrad/result_blast.html#sbp:Sbal223_2659) 1.7

[sbn:Sbal195_1748](http://www.genome.jp/dbget-bin/www_bget?sbn:Sbal195_1748) cold-shock DNA-binding domain-containing protei... [35](http://www.genome.jp/tmp/blast/120120152646PUrad/result_blast.html#sbn:Sbal195_1748) 1.7

[sbm:Shew185_1705](http://www.genome.jp/dbget-bin/www_bget?sbm:Shew185_1705) cold-shock DNA-binding domain-containing protei... [35](http://www.genome.jp/tmp/blast/120120152646PUrad/result_blast.html#sbm:Shew185_1705) 1.7

[sbl:Sbal_1708](http://www.genome.jp/dbget-bin/www_bget?sbl:Sbal_1708) cold-shock DNA-binding domain-containing protein; ... [35](http://www.genome.jp/tmp/blast/120120152646PUrad/result_blast.html#sbl:Sbal_1708) 1.7

[son:SO_2787](http://www.genome.jp/dbget-bin/www_bget?son:SO_2787) cold shock domain-contain protein; K03704 cold shock... [35](http://www.genome.jp/tmp/blast/120120152646PUrad/result_blast.html#son:SO_2787) 1.7

[avr:B565_2628](http://www.genome.jp/dbget-bin/www_bget?avr:B565_2628) cold shock-like protein CspE; K03704 cold shock pr... [35](http://www.genome.jp/tmp/blast/120120152646PUrad/result_blast.html#avr:B565_2628) 1.7

[saz:Sama_1970](http://www.genome.jp/dbget-bin/www_bget?saz:Sama_1970) cold-shock DNA-binding domain-containing protein; ... [35](http://www.genome.jp/tmp/blast/120120152646PUrad/result_blast.html#saz:Sama_1970) 1.8

[van:VAA_00964](http://www.genome.jp/dbget-bin/www_bget?van:VAA_00964) Cold shock protein; K03704 cold shock protein (bet... [35](http://www.genome.jp/tmp/blast/120120152646PUrad/result_blast.html#van:VAA_00964) 1.9

[kko:Kkor_0968](http://www.genome.jp/dbget-bin/www_bget?kko:Kkor_0968) cold-shock DNA-binding domain-containing protein; ... [34](http://www.genome.jp/tmp/blast/120120152646PUrad/result_blast.html#kko:Kkor_0968) 2.1

[mme:Marme_2702](http://www.genome.jp/dbget-bin/www_bget?mme:Marme_2702) cold-shock DNA-binding domain-containing protein;... [34](http://www.genome.jp/tmp/blast/120120152646PUrad/result_blast.html#mme:Marme_2702) 2.4

[mgm:Mmc1_3079](http://www.genome.jp/dbget-bin/www_bget?mgm:Mmc1_3079) cold-shock DNA-binding protein family protein; K03... [34](http://www.genome.jp/tmp/blast/120120152646PUrad/result_blast.html#mgm:Mmc1_3079) 2.7

[pmk:MDS_3347](http://www.genome.jp/dbget-bin/www_bget?pmk:MDS_3347) cold-shock DNA-binding protein family protein; K037... [34](http://www.genome.jp/tmp/blast/120120152646PUrad/result_blast.html#pmk:MDS_3347) 2.8

[gni:GNIT_3404](http://www.genome.jp/dbget-bin/www_bget?gni:GNIT_3404) cspA; cold-shock DNA-binding domain-containing pro... [34](http://www.genome.jp/tmp/blast/120120152646PUrad/result_blast.html#gni:GNIT_3404) 2.9

[spe:Spro_1189](http://www.genome.jp/dbget-bin/www_bget?spe:Spro_1189) cspE; cold shock protein CspE; K03704 cold shock p... [34](http://www.genome.jp/tmp/blast/120120152646PUrad/result_blast.html#spe:Spro_1189) 3.0

[ttu:TERTU_3792](http://www.genome.jp/dbget-bin/www_bget?ttu:TERTU_3792) cold shock DNA-binding domain-containing protein;... [34](http://www.genome.jp/tmp/blast/120120152646PUrad/result_blast.html#ttu:TERTU_3792) 3.1

[xne:XNC1_1321](http://www.genome.jp/dbget-bin/www_bget?xne:XNC1_1321) cspE; RNA chaperone, transcription antiterminator,... [33](http://www.genome.jp/tmp/blast/120120152646PUrad/result_blast.html#xne:XNC1_1321) 3.4

[acd:AOLE_05855](http://www.genome.jp/dbget-bin/www_bget?acd:AOLE_05855) cold shock-like protein CspG; K03704 cold shock p... [33](http://www.genome.jp/tmp/blast/120120152646PUrad/result_blast.html#acd:AOLE_05855) 3.5

[mmw:Mmwyl1_1655](http://www.genome.jp/dbget-bin/www_bget?mmw:Mmwyl1_1655) cold-shock DNA-binding domain-containing protein... [33](http://www.genome.jp/tmp/blast/120120152646PUrad/result_blast.html#mmw:Mmwyl1_1655) 3.5

[pmy:Pmen_3069](http://www.genome.jp/dbget-bin/www_bget?pmy:Pmen_3069) cold-shock DNA-binding protein family protein; K03... [33](http://www.genome.jp/tmp/blast/120120152646PUrad/result_blast.html#pmy:Pmen_3069) 3.6

[sgl:SG0145](http://www.genome.jp/dbget-bin/www_bget?sgl:SG0145) cold-shock protein; K03704 cold shock protein (beta-r... [33](http://www.genome.jp/tmp/blast/120120152646PUrad/result_blast.html#sgl:SG0145) 3.7

[men:MEPCIT_050](http://www.genome.jp/dbget-bin/www_bget?men:MEPCIT_050) cspD; putative major cold shock protein; K03704 c... [33](http://www.genome.jp/tmp/blast/120120152646PUrad/result_blast.html#men:MEPCIT_050) 3.8

[avr:B565_0421](http://www.genome.jp/dbget-bin/www_bget?avr:B565_0421) cold shock-like protein CspI; K03704 cold shock pr... [33](http://www.genome.jp/tmp/blast/120120152646PUrad/result_blast.html#avr:B565_0421) 3.8

[asa:ASA_4001](http://www.genome.jp/dbget-bin/www_bget?asa:ASA_4001) cspA; major cold shock protein; K03704 cold shock p... [33](http://www.genome.jp/tmp/blast/120120152646PUrad/result_blast.html#asa:ASA_4001) 3.8

[dal:Dalk_2720](http://www.genome.jp/dbget-bin/www_bget?dal:Dalk_2720) cold-shock DNA-binding domain-containing protein; ... [33](http://www.genome.jp/tmp/blast/120120152646PUrad/result_blast.html#dal:Dalk_2720) 4.0

[aha:AHA_3962](http://www.genome.jp/dbget-bin/www_bget?aha:AHA_3962) hypothetical protein; K03704 cold shock protein (be... [33](http://www.genome.jp/tmp/blast/120120152646PUrad/result_blast.html#aha:AHA_3962) 4.2

[vha:VIBHAR_05396](http://www.genome.jp/dbget-bin/www_bget?vha:VIBHAR_05396) hypothetical protein; K03704 cold shock protein... [33](http://www.genome.jp/tmp/blast/120120152646PUrad/result_blast.html#vha:VIBHAR_05396) 4.4

[maq:Maqu_1731](http://www.genome.jp/dbget-bin/www_bget?maq:Maqu_1731) cold-shock DNA-binding domain-containing protein; ... [33](http://www.genome.jp/tmp/blast/120120152646PUrad/result_blast.html#maq:Maqu_1731) 4.5

[ppr:PBPRB1779](http://www.genome.jp/dbget-bin/www_bget?ppr:PBPRB1779) cold shock protein [33](http://www.genome.jp/tmp/blast/120120152646PUrad/result_blast.html#ppr:PBPRB1779) 4.9

[mpc:Mar181_0682](http://www.genome.jp/dbget-bin/www_bget?mpc:Mar181_0682) cold-shock DNA-binding domain-containing protein... [33](http://www.genome.jp/tmp/blast/120120152646PUrad/result_blast.html#mpc:Mar181_0682) 5.1

[neu:NE1312](http://www.genome.jp/dbget-bin/www_bget?neu:NE1312) cspD2; cold shock DNA-binding domain-containing prote... [33](http://www.genome.jp/tmp/blast/120120152646PUrad/result_blast.html#neu:NE1312) 5.2

[pmr:PMI0418](http://www.genome.jp/dbget-bin/www_bget?pmr:PMI0418) cspE; cold shock protein CspE; K03704 cold shock pro... [33](http://www.genome.jp/tmp/blast/120120152646PUrad/result_blast.html#pmr:PMI0418) 5.3

[mmw:Mmwyl1_3621](http://www.genome.jp/dbget-bin/www_bget?mmw:Mmwyl1_3621) cold-shock DNA-binding domain-containing protein... [33](http://www.genome.jp/tmp/blast/120120152646PUrad/result_blast.html#mmw:Mmwyl1_3621) 5.5

[psz:PSTAB_2678](http://www.genome.jp/dbget-bin/www_bget?psz:PSTAB_2678) cspA; cold shock protein CspA; K03704 cold shock ... [33](http://www.genome.jp/tmp/blast/120120152646PUrad/result_blast.html#psz:PSTAB_2678) 5.6

[psa:PST_2693](http://www.genome.jp/dbget-bin/www_bget?psa:PST_2693) cold shock protein CspA; K03704 cold shock protein ... [33](http://www.genome.jp/tmp/blast/120120152646PUrad/result_blast.html#psa:PST_2693) 5.7

[pag:PLES_04521](http://www.genome.jp/dbget-bin/www_bget?pag:PLES_04521) putative cold-shock protein; K03704 cold shock pr... [33](http://www.genome.jp/tmp/blast/120120152646PUrad/result_blast.html#pag:PLES_04521) 5.9

[pap:PSPA7_0559](http://www.genome.jp/dbget-bin/www_bget?pap:PSPA7_0559) putative cold-shock protein; K03704 cold shock pr... [33](http://www.genome.jp/tmp/blast/120120152646PUrad/result_blast.html#pap:PSPA7_0559) 5.9

[pau:PA14_05960](http://www.genome.jp/dbget-bin/www_bget?pau:PA14_05960) cspB; cold-shock protein; K03704 cold shock prote... [33](http://www.genome.jp/tmp/blast/120120152646PUrad/result_blast.html#pau:PA14_05960) 5.9

[pae:PA0456](http://www.genome.jp/dbget-bin/www_bget?pae:PA0456) cold-shock protein; K03704 cold shock protein (beta-r... [33](http://www.genome.jp/tmp/blast/120120152646PUrad/result_blast.html#pae:PA0456) 5.9

[vex:VEA_001460](http://www.genome.jp/dbget-bin/www_bget?vex:VEA_001460) cold shock protein CspE; K03704 cold shock protei... [33](http://www.genome.jp/tmp/blast/120120152646PUrad/result_blast.html#vex:VEA_001460) 5.9

[Show all result](http://www.genome.jp/tmp/blast/120120152646PUrad/result_blast.html)

Bottom of Form

[DBGET](http://www.genome.jp/dbget/) integrated database retrieval system, [GenomeNet](http://www.genome.jp/)

(Out of the 202 hits obtained the 100 hits are shown above). The list includes some organisms in the same genera)

5. Blast search of KEGG genome database of *Halomonas elongata for homologue of 8 kDa protein*

## BLASTP Search Result *Halomonas eurihalina* 8 kDa cold shock protein

**Database: hel**

**Protein sequence database entries related to query** - 4 hits

Top of Form

command: blastp

query: /share/www/blast/1201201525406UTf2/query.seq

database: h.elongata.pep (prot)

------------------------

command query database

blastp prot prot

Entry bits E-val

--------------------------------------------------------------------- -----------

[hel:HELO_3240](http://www.genome.jp/dbget-bin/www_bget?hel:HELO_3240) csp; cold shock protein CspA; K03704 cold shock pr... [30](http://www.genome.jp/tmp/blast/1201201525406UTf2/result_blast.html#hel:HELO_3240) 0.030

[hel:HELO_3431](http://www.genome.jp/dbget-bin/www_bget?hel:HELO_3431) csp; cold shock protein CspA; K03704 cold shock pr... [29](http://www.genome.jp/tmp/blast/1201201525406UTf2/result_blast.html#hel:HELO_3431) 0.050

[hel:HELO_1644](http://www.genome.jp/dbget-bin/www_bget?hel:HELO_1644) csp; cold shock protein CspA; K03704 cold shock pr... [28](http://www.genome.jp/tmp/blast/1201201525406UTf2/result_blast.html#hel:HELO_1644) 0.060

[hel:HELO_3812](http://www.genome.jp/dbget-bin/www_bget?hel:HELO_3812) cold shock protein CspA; K03704 cold shock protein... [27](http://www.genome.jp/tmp/blast/1201201525406UTf2/result_blast.html#hel:HELO_3812) 0.14

[Show all result](http://www.genome.jp/tmp/blast/1201201525406UTf2/result_blast.html)

Bottom of Form

[DBGET](http://www.genome.jp/dbget/) integrated database retrieval system, [GenomeNet](http://www.genome.jp/)

6. ClustalW2 Results

CLUSTAL 2.1 multiple sequence alignment 8kDa cold shock protein (query)

**hel_HELO_3240 ------------------------------------------------------------**

**hel_HELO_3431 ------------------------------------------------------------**

**hel_HELO_1644 ------------------------------------------------------------**

**query ------------------------------------------------------------**

**hel_HELO_3812 MNRKVVLRCSLISLLLAAPSPLLVALFVHLAGGAMSREIFASLEIGGVGAVYLAVALAVF 60**

**hel_HELO_3240 ---------------------------MATGTVKWFNDTKGFGFISPDDNGDDLFAHFSE 33**

**hel_HELO_3431 ---------------------------MATGTVKWFNDTKGYGFISPEDGGDDLFVHFSE 33**

**hel_HELO_1644 ---------------------------MTTGTVKWFNDSKGFGFISPADGGDDVFAHFSE 33**

**query ------------------------------------------------------------**

**hel_HELO_3812 VLLLIATLAVNALTPQLVNLAEVEDDDREIGEVKWFNVNKGYGFITR-DSGEDVFVHFRA 119**

**hel_HELO_3240 IQAEGFKSLQDGQKVSFDVTQGKKGLQASNIKVVD 68**

**hel_HELO_3431 IQAEGFKSLQDGQKVSFEVTQGKKGLQASNVRVTD 68**

**hel_HELO_1644 IQAEGFKSLQEGQSVSFDVTQGKKGLQASNIKALG 68**

**query ------KTLAEGQKVEFTVTQGQKG---------- 19**

**hel_HELO_3812 IRGRGHRTLAEGQKVKYQVSRNERGLQADDVTVIT 154**

**::* :**.*.: *::.::***


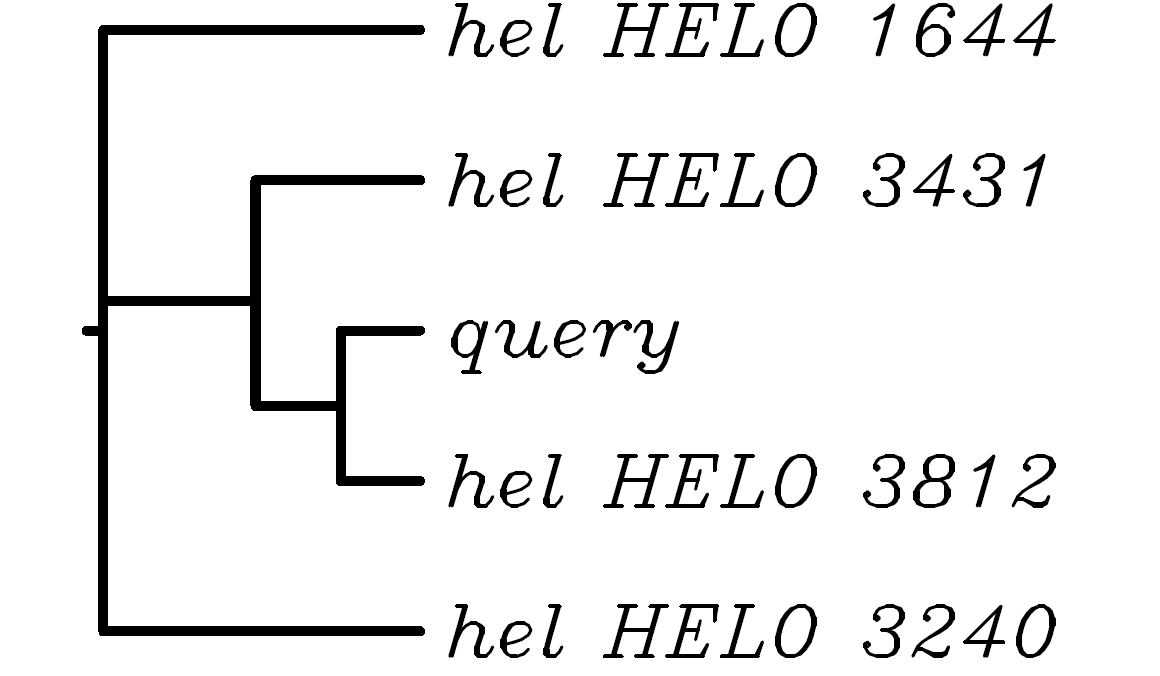


Rooted phylogenetic tree (neighbor joining) of 8 kDa protein showing similarity to HELO_3812.

7. ClustalW2 Results

CLUSTAL 2.1 multiple sequence alignment 8 kDa cold shock protein excluding hel_HELO 3812 (since its molecular weight is very high compared to 8 kDa protein)

**hel_HELO_3240 MATGTVKWFNDTKGFGFISPDDNGDDLFAHFSEIQAEGFKSLQDGQKVSFDVTQGKKGLQ 60**

**hel_HELO_3431 MATGTVKWFNDTKGYGFISPEDGGDDLFVHFSEIQAEGFKSLQDGQKVSFEVTQGKKGLQ 60**

**hel_HELO_1644 MTTGTVKWFNDSKGFGFISPADGGDDVFAHFSEIQAEGFKSLQEGQSVSFDVTQGKKGLQ 60**

**query ---------------------------------------KTLAEGQKVEFTVTQGQKG-- 19**

***:* :**.*.* ****:****

**hel_HELO_3240 ASNIKVVD 68**

**hel_HELO_3431 ASNVRVTD 68**

**hel_HELO_1644 ASNIKALG 68**

**query --------**

**
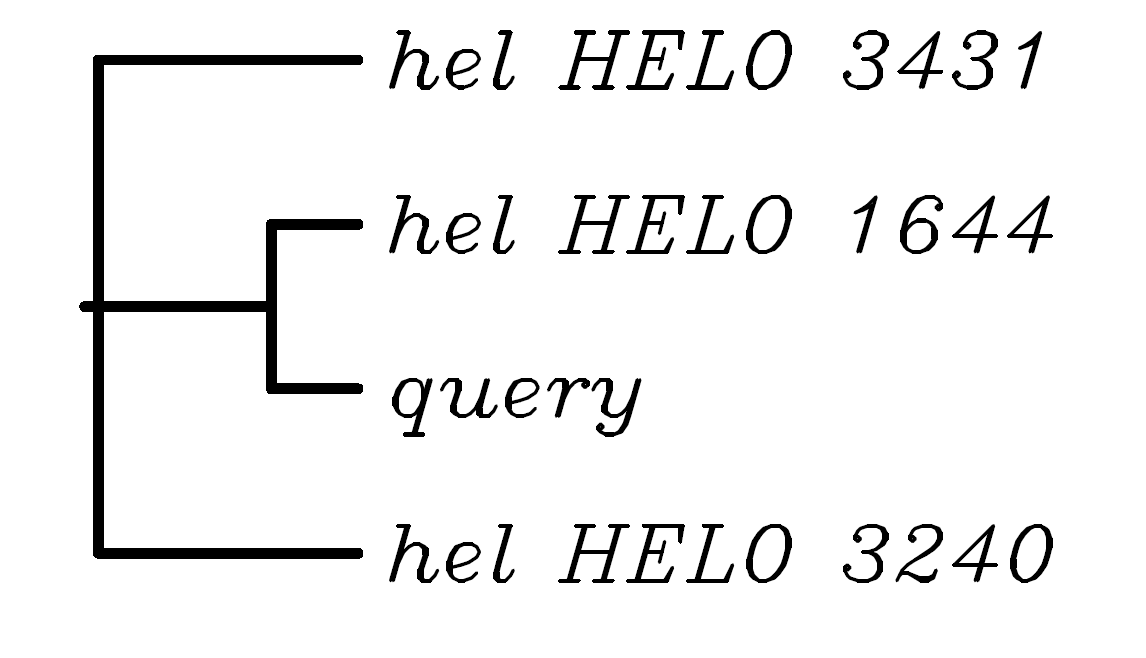
**

Rooted phylogenetic tree (neighbor joining) of 8 kDa protein (excluding HELO_3812) showing similarity to HELO_1644
